# Hypoglycaemia frequency and physiological response after double or triple doses of once-weekly insulin icodec vs once-daily insulin glargine U100 in type 2 diabetes: a randomised crossover trial

**DOI:** 10.1007/s00125-023-05921-8

**Published:** 2023-06-13

**Authors:** Thomas R. Pieber, Kristine N. Arfelt, Roman Cailleteau, Marlies Hart, Soumitra Kar, Ines Mursic, Eva Svehlikova, Martina Urschitz, Hanne Haahr

**Affiliations:** 1 Department of Internal Medicine, Medical University of Graz, Graz, Austria; 2 Novo Nordisk A/S, Søborg, Denmark; 3 Novo Nordisk Service Centre India Private Ltd., Bangalore, India

**Keywords:** Cognitive function, Counterregulation, Hypoglycaemia, Hypoglycaemia awareness, Hypoglycaemic symptoms, Insulin glargine U100, Insulin icodec, Once-weekly, Pharmacokinetics, Type 2 diabetes

## Abstract

**Aims/hypothesis:**

This study compared the frequency of hypoglycaemia, time to hypoglycaemia and recovery from hypoglycaemia after double or triple doses of once-weekly insulin icodec vs once-daily insulin glargine U100. Furthermore, the symptomatic and counterregulatory responses to hypoglycaemia were compared between icodec and glargine U100 treatment.

**Methods:**

In a randomised, single-centre (Department of Internal Medicine, Division of Endocrinology and Diabetology, Medical University of Graz, Graz, Austria), open-label, two-period crossover trial, individuals with type 2 diabetes (age 18–72 years, BMI 18.5–37.9 kg/m^2^, HbA_1c_ ≤75 mmol/mol [≤9.0%]) treated with basal insulin with or without oral glucose-lowering drugs received once-weekly icodec (for 6 weeks) and once-daily glargine U100 (for 11 days). Total weekly doses were equimolar based on individual titration of daily glargine U100 during the run-in period (target fasting plasma glucose [PG]: 4.4–7.2 mmol/l). Randomisation was carried out by assigning a randomisation number to each participant in ascending order, which encoded to one of two treatment sequences via a randomisation list prepared prior to the start of the trial. At steady state, double and triple doses of icodec and glargine U100 were administered followed by hypoglycaemia induction: first, euglycaemia was maintained at 5.5 mmol/l by variable i.v. infusion of glucose; glucose infusion was then terminated, allowing PG to decrease to no less than 2.5 mmol/l (target PG_nadir_). The PG_nadir_ was maintained for 15 min. Euglycaemia was restored by constant i.v. glucose (5.5 mg kg^−1^ min^−1^). Hypoglycaemic symptoms score (HSS), counterregulatory hormones, vital signs and cognitive function were assessed at predefined PG levels towards the PG_nadir_.

**Results:**

Hypoglycaemia induction was initiated in 43 and 42 participants after double dose of icodec and glargine U100, respectively, and in 38 and 40 participants after triple doses, respectively. Clinically significant hypoglycaemia, defined as PG_nadir_ <3.0 mmol/l, occurred in comparable proportions of individuals treated with icodec vs glargine U100 after double (17 [39.5%] vs 15 [35.7%]; *p*=0.63) and triple (20 [52.6%] vs 28 [70.0%]; *p*=0.14) doses. No statistically significant treatment differences were observed in the time to decline from PG values of 5.5 mmol/l to 3.0 mmol/l (2.9–4.5 h after double dose and 2.2–2.4 h after triple dose of the insulin products). The proportion of participants with PG_nadir_ ≤2.5 mmol/l was comparable between treatments after double dose (2 [4.7%] for icodec vs 3 [7.1%] for glargine U100; *p*=0.63) but higher for glargine U100 after triple dose (1 [2.6%] vs 10 [25.0%]; *p*=0.03). Recovery from hypoglycaemia by constant i.v. glucose infusion took <30 min for all treatments. Analyses of the physiological response to hypoglycaemia only included data from participants with PG_nadir_ <3.0 mmol/l and/or the presence of hypoglycaemic symptoms; in total 20 (46.5%) and 19 (45.2%) individuals were included after a double dose of icodec and glargine U100, respectively, and 20 (52.6%) and 29 (72.5%) individuals were included after a triple dose of icodec and glargine U100, respectively. All counterregulatory hormones (glucagon, adrenaline [epinephrine], noradrenaline [norepinephrine], cortisol and growth hormone) increased during hypoglycaemia induction with both insulin products at both doses. Following triple doses, the hormone response was greater with icodec vs glargine U100 for adrenaline at PG_3.0 mmol/l_ (treatment ratio 2.54 [95% CI 1.69, 3.82]; *p*<0.001), and cortisol at PG_3.0_ _mmol/l_ (treatment ratio 1.64 [95% CI 1.13, 2.38]; *p*=0.01) and PG_nadir_ (treatment ratio 1.80 [95% CI 1.09, 2.97]; *p*=0.02). There were no statistically significant treatment differences in the HSS, vital signs and cognitive function.

**Conclusions/interpretation:**

Double or triple doses of once-weekly icodec lead to a similar risk of hypoglycaemia compared with double or triple doses of once-daily glargine U100. During hypoglycaemia, comparable symptomatic and moderately greater endocrine responses are elicited by icodec vs glargine U100.

**Trial registration:**

ClinicalTrials.gov NCT03945656.

**Funding:**

This study was funded by Novo Nordisk A/S.

**Graphical Abstract:**

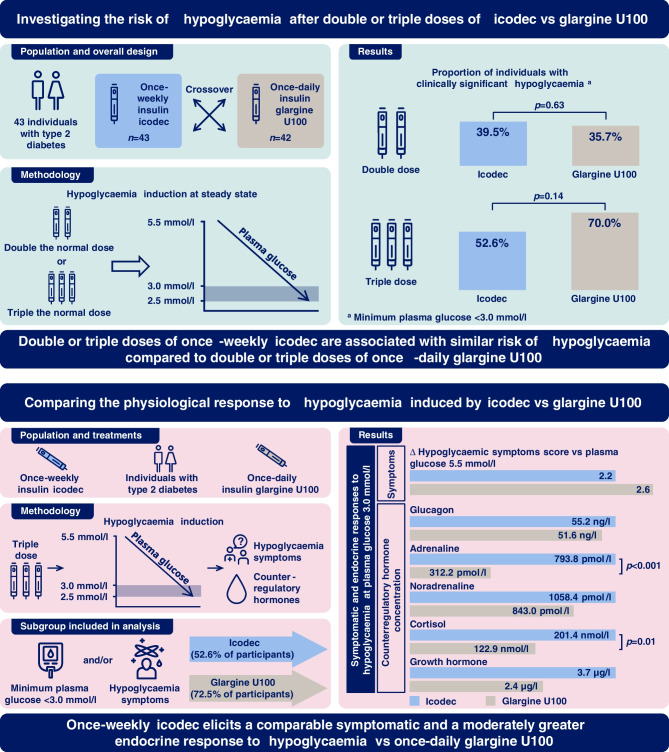

**Supplementary Information:**

The online version contains peer-reviewed but unedited supplementary material available at 10.1007/s00125-023-05921-8.


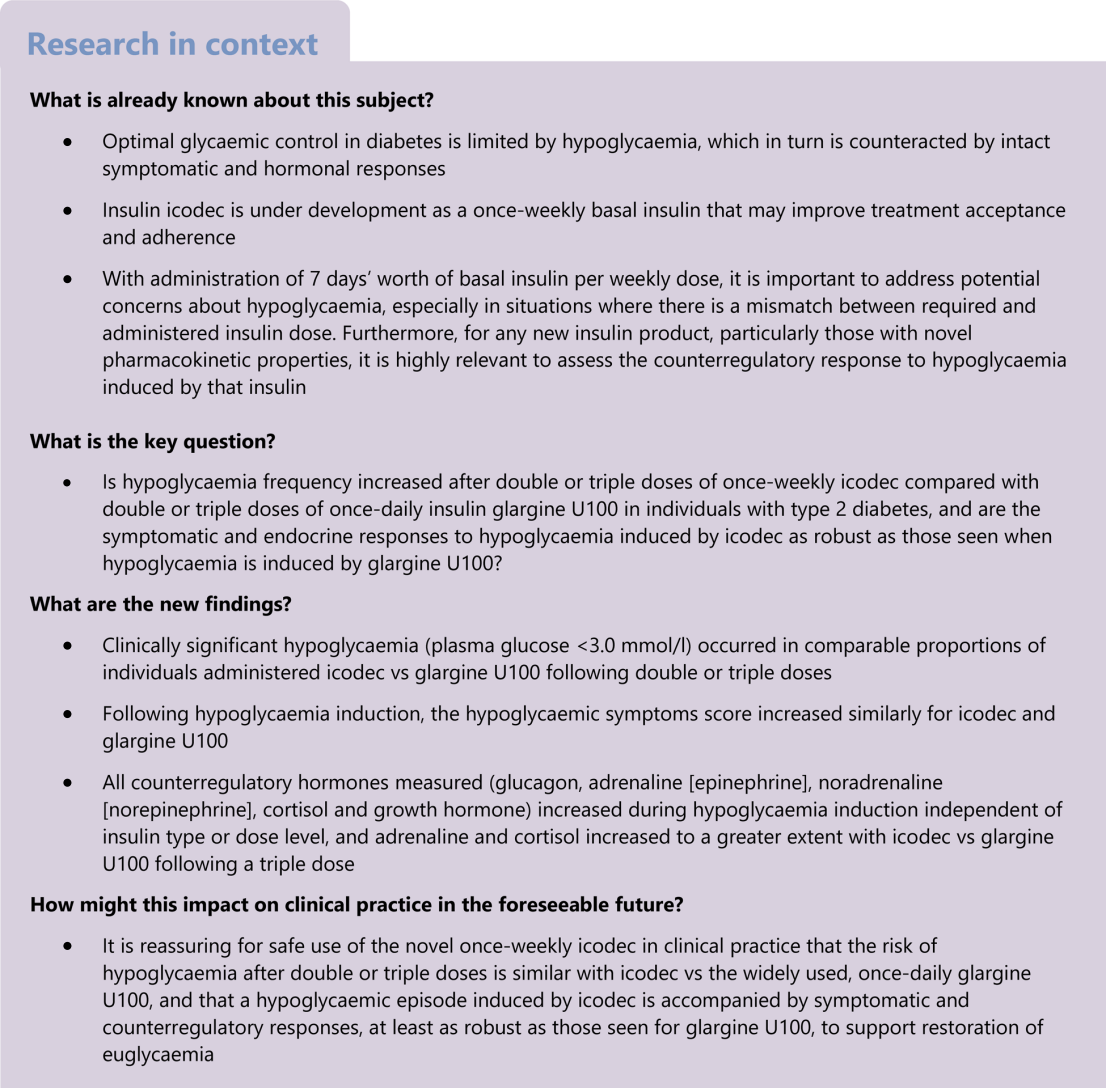


## Introduction

Despite ongoing advances in diabetes therapy, hypoglycaemia and the fear of hypoglycaemia are still considerable barriers for optimal glycaemic control in diabetes [1–3]. Compared with the current daily basal insulin treatments, once-weekly insulin could potentially relieve therapeutic inertia and improve treatment adherence by simplifying insulin therapy [4]. However, with weekly insulin administration, it is necessary to address potential concerns about hypoglycaemia, particularly related to unintentional mismatches between insulin requirement and dose administered, for example in the case of a miscalculated dose.

In type 2 diabetes, longer duration of diabetes and increased time since initiation of insulin therapy are both associated with diminishing hypoglycaemia awareness and blunted counterregulation [5, 6]. This may at least partly explain why the risk of hypoglycaemia increases with type 2 diabetes disease progression [7]. Novel insulin products should not be associated with a further deterioration of hypoglycaemia awareness and counterregulation. For any newly developed basal insulin, it is, therefore, important to investigate whether hypoglycaemia induced by that insulin elicits a robust symptomatic and counterregulatory response. It was previously shown that once-daily basal insulin degludec and insulin glargine U100 provided significant symptomatic, endocrine and cognitive responses to induced hypoglycaemia [8].

Insulin icodec is a basal insulin that is being developed for once-weekly administration [9]. Following s.c. injection, icodec is absorbed into the circulation, where it binds strongly and reversibly to albumin creating an essentially inactive depot from which icodec is slowly and continuously released providing a half-life suitable for once-weekly dosing [10]. Activation of insulin receptor-mediated signalling by icodec and the subsequent metabolic response is similar to that of native human insulin [10]. Phase 2 trials have shown similar reductions in HbA_1c_ and fasting plasma glucose (PG), and comparable rates of level 2 and level 3 hypoglycaemia, with once-weekly icodec vs once-daily glargine U100 in type 2 diabetes [11–13]. Importantly, the duration of hypoglycaemic episodes was similar for once-weekly icodec relative to once-daily glargine U100 (R. J. Silver, New York Presbyterian-Brooklyn Methodist Hospital, NY, USA, unpublished results).

As icodec is a novel basal insulin, the glucodynamics, counterregulation and safety associated with hypoglycaemia require further characterisation. This trial investigated hypoglycaemia frequency, time to hypoglycaemia, recovery from hypoglycaemia and overall safety after double or triple doses of once-weekly icodec vs once-daily glargine U100 in type 2 diabetes. Furthermore, symptomatic and hormonal counterregulatory responses to hypoglycaemia were compared between icodec and glargine U100 in a subgroup analysis that only included individuals when they actually experienced hypoglycaemia after a double or triple dose of either insulin product. To our knowledge, this is the first study to investigate the symptomatic and endocrine responses to hypoglycaemia induced by a basal insulin analogue in individuals with type 2 diabetes.

## Methods

### Research design

This was a randomised, single-centre (Department of Internal Medicine, Division of Endocrinology and Diabetology, Medical University of Graz, Graz, Austria), open-label, two-period crossover trial (ClinicalTrials.gov registration no. NCT03945656). The trial was conducted to investigate hypoglycaemia frequency and the response to hypoglycaemia following icodec and glargine U100 administration at double and triple dose levels. Hypoglycaemia frequency was assessed based on all participants, while the response to hypoglycaemia was assessed in a subgroup analysis based on individuals who actually experienced hypoglycaemia after a double or triple insulin dose, i.e. those with a PG decline to <3.0 mmol/l and/or the presence of hypoglycaemic symptoms.

The protocol was reviewed and approved by appropriate health authorities according to local regulation, and by the Independent Ethics Committee of the Medical University of Graz. The trial was performed in accordance with the Declaration of Helsinki and Good Clinical Practice. Prior to any trial-related activities, participants provided written informed consent.

### Participants

Potential participants were recruited based on the site’s database of individuals with type 2 diabetes, advertisements in printed and electronic media including social media reaching the broad Austrian population, and referrals from general practitioners and specialists in internal medicine.

Eligible participants were men or women aged 18–72 years, diagnosed with type 2 diabetes for ≥180 days before screening, treated with basal insulin (total daily insulin dose of 0.2–1.0 U/kg) with or without oral glucose-lowering drugs (metformin, sulfonylureas, glinides, dipeptidyl peptidase-4 inhibitors and sodium–glucose cotransporter 2 inhibitors were allowed) ≥90 days before screening, with a BMI of 18.5–37.9 kg/m^2^, HbA_1c_ ≤75 mmol/mol (≤9.0%), and supine blood pressure 90–159 mmHg (systolic) and 50–99 mmHg (diastolic). Individuals with recurrent severe hypoglycaemia (>1 severe hypoglycaemic episode ≤180 days before screening), hypoglycaemia unawareness, or previous or current cardiovascular disease, and those who had been hospitalised for diabetic ketoacidosis ≤180 days before screening, smokers and pregnant women were excluded from participation. Sex and race were determined by asking the participants.

Based on the eligibility criteria, the trial population was a selection of the broad type 2 diabetes population, i.e. being treated with basal insulin with or without oral glucose-lowering drugs and with no clinically significant diabetes complications and no history of cardiovascular disease. The latter criterion was chosen due to the burdensome nature of the trial owing to the induced hypoglycaemia element of the study. Thus, the participant selection was balanced between including the target population of basal insulin users and assuring an acceptable risk with the trial intervention. The enrolled participants were considered representative of the target trial population with respect to sex, age and race.

### Procedures and assessments

**Fig. 1:**
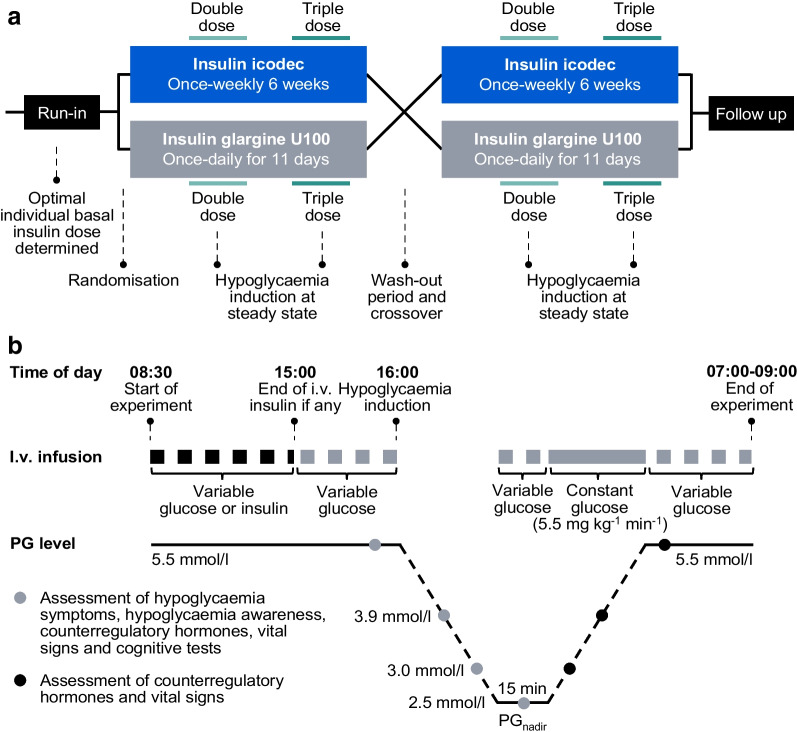
(**a**) Overall trial design and (**b**) hypoglycaemia induction methodology

The trial comprised a screening visit, an oral glucose-lowering drug washout period, a run-in period, two treatment periods and a follow-up visit (Fig. 1a).

#### Washout period

The oral glucose-lowering drug washout period (≥14 days) applied only to participants entering the trial on oral glucose-lowering drugs other than metformin. Metformin treatment continued throughout the trial at a stable dose.

#### Run-in period

During the run-in period (3–49 days), participants received once-daily glargine U100 with or without any usual metformin to determine the individual basal glargine U100 dose titrated to a fasting self-measured PG target of 4.4–7.2 mmol/l. Randomisation was performed during the run-in period when the mean of 3 consecutive days’ fasting self-measured PG was within 4.4–7.2 mmol/l with stable glargine U100 dose. Each participant was assigned a randomisation number in ascending order at the clinical site, which encoded the participant’s assignment to one of two treatment sequences via a randomisation list prepared by the sponsor (Novo Nordisk A/S).

#### Treatment periods

During the two treatment periods, participants received once-weekly icodec for 6 weeks or once-daily glargine U100 for 11 days in randomised order. The difference in treatment duration between icodec and glargine U100 was due to the difference in dosing frequencies and, hence, time to steady state, since hypoglycaemia induction following double and triple doses was performed at steady state. Treatment periods were separated by 35–49 days of washout following icodec and 4–11 days of washout following glargine U100. Icodec (4200 nmol/ml; Novo Nordisk, Bagsværd, Denmark) was administered s.c. in the left thigh at 20:00 hours by qualified site staff using a NovoPen 4 pen injector (Novo Nordisk). A normal dose of once-weekly icodec was defined as seven times the individual basal once-daily glargine U100 dose established during run in; hence, the total weekly dose was equal for both insulin products. At the start of week 1, a one-time additional 100% icodec dose was administered to accelerate time to steady state, followed by a normal dose at week 2, a double dose at week 3 (prior to hypoglycaemia induction), no dose at week 4 (due to the double dose given the week before), a normal dose at week 5, and a triple dose at week 6 (prior to hypoglycaemia induction). Insulin glargine U100 (100 U/ml [600 nmol/ml]; Sanofi, Paris, France) was administered s.c. in the right thigh at 09:00 hours by qualified site staff using a SoloStar pen injector (Sanofi). A normal dose of once-daily glargine U100 was established during run in. Glargine U100 was administered at a normal dose for 3 days followed by a double dose on day 4 (prior to hypoglycaemia induction), no dose on day 5 (due to the double dose given the day before), then a normal dose for 5 days, and a triple dose on day 11 (prior to hypoglycaemia induction).

#### Follow-up visit

The follow-up visit occurred 39–45 days after icodec and 4–10 days after glargine U100 treatment.

#### Hypoglycaemia induction experiments

Hypoglycaemia induction was carried out during weeks 3 (double dose) and 6 (triple dose) for icodec and on days 4 (double dose) and 11 (triple dose) for glargine U100 while at steady state. To initiate hypoglycaemia induction, participants should not have experienced hypoglycaemia (PG <3.9 mmol/l) within 24 h or have any relevant medical condition that could confound the outcomes or pose unacceptable risk to the participant. Hypoglycaemia induction (Fig. 1b) was initiated at 16:00 hours 2 days after double/triple icodec dosing (44 h post dose) and on the day of double/triple glargine U100 dosing (7 h post dose). These timings were selected since they approximated the expected time of maximum glucose-lowering effect for both insulin products based on pharmacokinetic/pharmacodynamic modelling performed prior to the trial. For 7.5 h prior to hypoglycaemia induction, PG was kept at 5.5 mmol/l by variable i.v. infusion of glucose (20% wt/vol) or human soluble insulin (100 U/ml in 99.6 ml saline [154 mmol/l NaCl]). PG was measured by a Super GL2 Glucose Analyzer (Dr. Müller Gerätebau, Freital, Germany). Insulin infusion was terminated 60 min prior to hypoglycaemia induction at the latest. PG was maintained at 5.5 mmol/l±20% from 60 min to 30 min prior to hypoglycaemia induction and at 5.5 mmol/l±10% during the last 30 min prior to hypoglycaemia induction. Hypoglycaemia induction was initiated by terminating glucose infusion, thereby allowing PG to decline towards the minimum (nadir) PG target level of no less than 2.5 mmol/l. The handling of alternative scenarios, e.g. PG_nadir_ >2.5 mmol/l, slow PG decline towards hypoglycaemia or slow recovery from hypoglycaemia, is described in electronic supplementary material (ESM) Methods. The investigator recorded if the PG_nadir_ was <3.0 mmol/l or ≥3.0 mmol/l with hypoglycaemic symptoms. The PG_nadir_ level was maintained for 15 min by variable i.v. glucose as needed. Subsequently, euglycaemia was restored by constant i.v. glucose (5.5 mg kg^−1^ min^−1^) until PG was 5.5 mmol/l, which was maintained until the next morning (between 07:00 hours and 09:00 hours) by variable i.v. glucose. During hypoglycaemia induction, participants remained fasted (except for water ad libitum) and stayed in a supine or semi-supine position (except when cognitive tests were performed). Participants stayed at the clinical site for at least 12 h following each hypoglycaemia induction experiment. During this period, PG was monitored regularly, as deemed necessary by the investigator, and minor intake of rapidly absorbable carbohydrate was applied as needed to prevent PG values falling below 4.4 mmol/l. Participants were also advised to consume carbohydrate-rich meals and snacks as needed during the period after discharge from the clinical site to prevent hypoglycaemia.

Hypoglycaemic response assessments were conducted at predefined PG levels during hypoglycaemia development and recovery (Fig. 1b). Hypoglycaemic symptoms score (HSS), hypoglycaemia awareness, counterregulatory hormones, vital signs and cognitive function tests were assessed at baseline (PG_5.5 mmol/l_), during hypoglycaemia development (PG_3.9_ _mmol/l_ and PG_3.0 mmol/l_) and at PG_nadir_. In addition, counterregulatory hormones and vital signs were assessed during recovery from hypoglycaemia at PG_3.0 mmol/l_, PG_3.9_ _mmol/l_ and PG_5.5 mmol/l_.

#### HSS

The validated Edinburgh Hypoglycaemia Scale was used to determine hypoglycaemic symptoms, which were classified into 11 different autonomic, neuroglycopenic and non-specific symptoms of hypoglycaemia [14, 15]. The participant scored each of the 11 symptoms on a seven-point scale (1=‘not at all’, while 7=‘a great deal’). The total score was derived as 11 multiplied by the mean score for all symptoms.

#### Hypoglycaemia awareness

Hypoglycaemia awareness was determined by asking the question, ‘Do you feel hypo?’, to the participant.

#### Counterregulatory hormones

Plasma concentrations of glucagon were measured by an enzyme-linked immunosorbent assay (Mercodia AB, Uppsala, Sweden). Plasma concentrations of adrenaline (epinephrine) and noradrenaline (norepinephrine) were measured by liquid chromatography-mass spectrometry (Thermo Fisher Scientific, Dreieich, Germany). Serum concentrations of cortisol and growth hormone were measured by electrochemiluminescent immunoassays (Roche Diagnostics, Mannheim, Germany).

#### Vital signs

Vital signs (diastolic blood pressure, systolic blood pressure and pulse) were measured after ≥5 min of rest in a supine position.

#### Cognitive tests

Cognitive function was assessed using the trail making B (TMB) test, the digit symbol substitution test (DSST) and the four-choice reaction time (4CRT) test. In the TMB test from the Halstead Reitan Neuropsychological Battery [16, 17], the participant connects 25 circles distributed on a chart in the correct sequence as quickly as possible. The circles contain a number (1–13) or a letter (A–L), and the correct sequence consists of alternating numbers and letters (i.e. 1–A–2–B–3 etc). The test result is the time needed to complete the task correctly. In the DSST, which is a subtest of the Wechsler Adult Intelligence Scale - Revised [18], nine digits are represented by nine different symbols. During 90 s, the participant is requested to write down the appropriate symbol for as many numbers as possible in a given array. The test result is the number of correct responses. In the 4CRT test [19], a dot is randomly displayed in one of four possible fields of a computer screen. In 100 cycles, the participant responds by pressing the corresponding position on a keypad. The test result has two components: percentage of correct answers and the mean response time.

#### Pharmacokinetics

During the treatment periods, blood for assessment of icodec and glargine pharmacokinetics was sampled at predefined time points (ESM Tables 1 and 2) aimed at determining the exposure after a normal dose at steady state and after the double and triple doses. Serum icodec was measured using a validated icodec-specific immunoassay. Serum glargine was measured using a validated glargine-specific luminescent oxygen channelling immunoassay. Both assays were developed by Novo Nordisk.

#### Continuous glucose monitoring

In addition to self-measured PG, which was performed throughout the trial, and PG measurements performed at the clinical site, continuous glucose monitoring (CGM) was conducted from the first dose of the first treatment period until the end of the second treatment period (Dexcom G6; Dexcom, San Diego, CA, USA). CGM data were visible to the participants and the investigators but were not used for titration of basal insulin dose or for reporting hypoglycaemia.

#### Safety assessments

Safety assessments during the trial included adverse events (AEs), hypoglycaemic episodes (other than those induced), vital signs (other than those assessed during hypoglycaemia induction), electrocardiograms, physical examinations and clinical laboratory assessments (haematology, biochemistry and urinalysis).

### Endpoints and statistical analyses

Statistical analyses were conducted using SAS version 9.4 (SAS Institute, Cary, NC, USA). The significance level was set to 5%. No multiplicity adjustment was performed. Unless otherwise stated, the analyses included all randomised participants receiving ≥1 dose of the trial product. In the subgroup analysis to investigate the physiological response to hypoglycaemia, each participant only contributed to the analysis if a clinically relevant PG decline occurred and/or hypoglycaemic symptoms were present during the hypoglycaemia induction experiments. Thus, a participant was only included in the analysis for each dose level (double or triple) and insulin product if hypoglycaemia induction after that specific dose level/insulin product led to clinically significant hypoglycaemia (defined as minimum [nadir] PG <3.0 mmol/l) and/or hypoglycaemic symptoms as judged by the investigator.

The primary endpoint was clinically significant hypoglycaemia (PG_nadir_ <3.0 mmol/l) during hypoglycaemia induction after double dose of insulin. The sample size determination assumed that the proportion of participants who did not experience clinically significant hypoglycaemia after double dose of insulin was 35% for icodec and 10% for glargine U100, with a correlation coefficient (ρ) of 0.2 between treatments. Under these assumptions, 41 participants were required to detect a statistically significant treatment difference with >80% power. The proportion of participants with clinically significant hypoglycaemia after double dose of insulin was compared between icodec and glargine U100 using a logistic regression model with logit link that included treatment and period as fixed effects and participant as a random effect. If hypoglycaemia induction was terminated prematurely, prior to PG_nadir_, the primary endpoint was considered missing. The proportion of participants with clinically significant hypoglycaemia after triple dose of insulin and the proportion of participants with a PG_nadir_ ≤2.5 mmol/l after double and triple insulin doses were analysed in the same way as the proportion of participants who met the primary endpoint.

PG_nadir_ was log_*e*_-transformed and compared between icodec and glargine U100 at each dose level using a linear mixed-effects model with treatment and period as fixed effects and participant as a random effect. Results were back-transformed to the original scale, and estimated geometric means, treatment ratio and 95% CI were derived.

The duration of decline of PG during hypoglycaemia induction was assessed by the endpoints time to decline from PG_5.5 mmol/l_ to PG_3.9_ _mmol/l_ and time to decline from PG_5.5 mmol/l_ to PG_3.0_ _mmol/l_, which were determined in individuals with a PG decline to <3.0 mmol/l and/or hypoglycaemia symptoms. The time to recovery (time and glucose amount needed from PG_nadir_ to PG_5.5 mmol/l_) was determined in individuals with PG <3.3 mmol/l at initiation of recovery. All these endpoints were compared between icodec and glargine U100 at each dose level using the same approach as for PG_nadir_.

In the subgroup analysis, concentrations of counterregulatory hormones at PG_3.0 mmol/l_ during hypoglycaemia induction and at PG_nadir_ were compared between insulin products at each of the two dose levels using the same approach as described for PG_nadir_. As a sensitivity analysis, the comparisons at each dose level were also performed including only those participants with PG_nadir_ <3.0 mmol/l and/or symptoms of hypoglycaemia as judged by the investigator for both icodec and glargine U100. Changes in HSS, vital signs and cognitive function tests from baseline (PG_5.5 mmol/l_) to PG_3.0 mmol/l_ and to PG_nadir_ were compared between insulin products at each of the two dose levels using the same approach as described for PG_nadir_, except that no logarithmic transformation was done and that the baseline value was included as a covariate. Least-squares means, treatment difference and 95% CI were derived.

AEs and hypoglycaemic episodes (other than those induced) during the treatment periods were summarised. The end of each treatment period was defined as 7 days after the last icodec dose and 2 days after the last glargine U100 dose. Total participant-years of exposure for the treatment period was longer for icodec (4.8 years) than for glargine U100 (1.6 years). Hypoglycaemic episodes were classified as level 1 (PG <3.9 mmol/l and ≥3.0 mmol/l), level 2 (PG <3.0 mmol/l) and level 3 (severe cognitive impairment requiring assistance for recovery) [20].

### Pharmacokinetic modelling

Based on the observed serum icodec and serum glargine concentrations, pharmacokinetic modelling was used to compare exposure (AUC during one dosing interval [AUC_τ_]) and maximum concentration (C_max_) for double and triple doses vs a normal dose. Details on the pharmacokinetic models for icodec and glargine are provided in ESM Methods. For both insulin products, a nonlinear mixed-effects approach provided a set of model parameter values for each individual, from which the individual concentration–time profiles were derived. For each individual, AUC_τ_ and C_max_ were calculated for three different dosing intervals (normal, double and triple doses). Exposure ratios (double/normal, triple/normal) were then derived.

## Results

### Participants

The trial was conducted between 7 May 2019 and 27 September 2021. The participant disposition is shown in ESM Fig. 1. A total of 70 individuals were screened, 43 were randomised, 43 and 42 were exposed to icodec and glargine U100, respectively, and 42 completed the trial. Baseline characteristics for the 43 randomised individuals are shown in Table 1. Hypoglycaemia induction after double dose was initiated in 43 and 42 individuals for icodec and glargine U100, respectively. After triple dose, hypoglycaemia induction was initiated in 38 and 40 individuals for icodec and glargine U100, respectively.

**Table 1:** Baseline characteristics of full analysis set

Variable	Individuals with type 2 diabetes
*N*	43
Age, years	56.3 ± 9.1
Sex	
Men	31 (72.1)
Women	12 (27.9)
Race	
White	41 (95.3)
Asian	1 (2.3)
Asian Indian	1 (2.3)
Body weight, kg	87.1 ± 13.0
Height, m	1.75 ± 0.10
BMI, kg/m^2^	28.5 ± 3.6
HbA_1c_, mmol/mol	55.5 ± 7.9
HbA_1c_, %	7.2 ± 0.7
Fasting PG, mmol/l	7.3 ± 1.6
Fasting C-peptide, nmol/l	0.7 ± 0.6
Diabetes duration, years	13.5 ± 7.9
Any oral glucose-lowering drug at screening	41 (95.3)
Metformin at screening	38 (88.4)
Individual once-daily basal insulin dose, U/kg	0.35 ± 0.15

Data are mean±SD or *n *(%)

### Pharmacokinetics

**Fig. 2:**
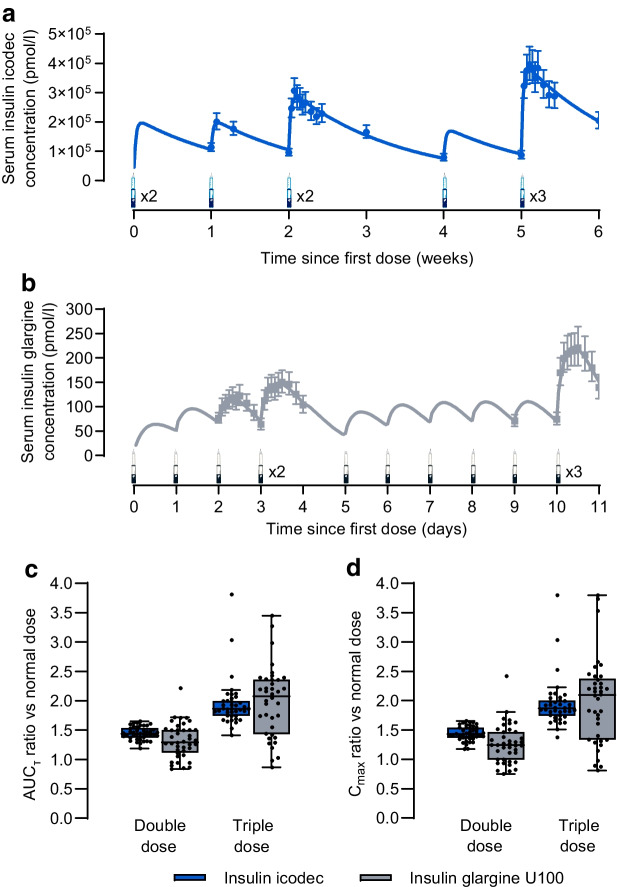
(**a**, **b**) Pharmacokinetic profiles of insulin icodec (**a**) and insulin glargine U100 (**b**) shown as observed values and simulated profiles using pharmacokinetic modelling. Observed values are geometric means and 95% CI (*n*=39 for icodec; *n*=38 for glargine U100). (**c**, **d**) Exposure (**c**) and C_max_ (**d**) ratios for double and triple doses vs normal dose of insulin based on the simulated pharmacokinetic profiles. Data are presented as median (middle horizontal line), interquartile range (top to bottom of box), minimum/maximum (upper/lower whisker excluding outlier values) and individual values (circles). Values lying more than 1.5 times the interquartile range from the box are excluded from the whiskers and shown as individual values only. Double dose: icodec, *n*=42; glargine U100, *n*=40. Triple dose: icodec, *n*=39; glargine U100, *n*=38

The observed concentrations and simulated pharmacokinetic profiles for icodec and glargine U100 reflected the dosing regimens, including administration of double and triple doses after achievement of steady state (Fig. 2a, b). As expected, the double and triple doses appeared to result in increasingly greater insulin exposure and C_max_ relative to a normal dose (Fig. 2c, d). The inter-individual variability in Fig. 2c, d appeared to be smaller for icodec than for glargine U100, suggesting that the exposure after double and triple doses relative to a normal dose was more consistent for icodec vs glargine U100.

### Hypoglycaemia development

**Fig. 3:**
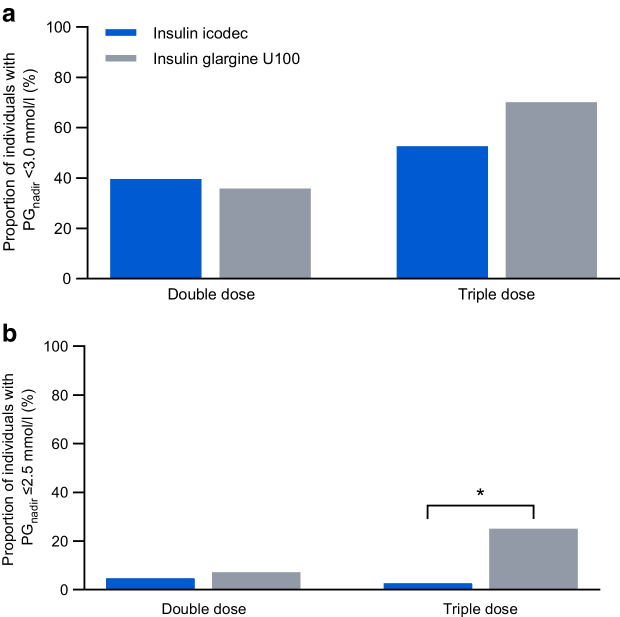
(**a**) Proportion of individuals with clinically significant hypoglycaemia defined as PG_nadir_ <3.0 mmol/l following double or triple doses of insulin icodec vs insulin glargine U100. Odds ratios (95% CI) for icodec/glargine U100 were 1.28 (0.46, 3.52) for double dose (*p*=0.63) and 0.48 (0.18, 1.28) for triple dose (*p*=0.14). (**b**) Proportion of individuals with PG_nadir_ ≤2.5 mmol/l following double or triple doses of icodec vs glargine U100. Odds ratios (95% CI) for icodec/glargine U100 were 0.62 (0.09, 4.40) for double dose (*p*=0.63) and 0.08 (0.01, 0.77) for triple dose (*p*=0.03). Double dose: icodec, *n*=43; glargine U100, *n*=42. Triple dose: icodec, *n*=38; glargine U100, *n*=40. *p<0.05, derived from two-sided test of no difference between icodec and glargine U100

Comparable proportions of individuals on icodec and glargine U100 experienced clinically significant hypoglycaemia (PG_nadir_ <3.0 mmol/l) following both double (*n*=17/43 [39.5%] and *n*=15/42 [35.7%], respectively; odds ratio icodec/glargine U100 [95% CI] 1.28 [0.46, 3.52]; *p*=0.63) and triple (*n*=20/38 [52.6%] and *n*=28/40 [70.0%], respectively; odds ratio [95% CI] 0.48 [0.18, 1.28]; *p*=0.14) doses (Fig. 3a). The proportion of individuals with PG_nadir_ ≤2.5 mmol/l after double dose of insulin was low for icodec and glargine U100 (*n*=2 [4.7%] and *n*=3 [7.1%], respectively; odds ratio icodec/glargine U100 [95% CI] 0.62 [0.09, 4.40]; *p*=0.63; Fig. 3b). After triple doses, it was still low for icodec (*n*=1 [2.6%]) but it was statistically significantly higher for glargine U100 (*n*=10 [25.0%]; odds ratio [95% CI] 0.08 [0.01, 0.77]; *p*=0.03). Accordingly, mean PG_nadir_ was comparable between treatments after double dose, while it was slightly but statistically significantly higher for icodec vs glargine U100 after triple dose (Table 2). The decline in PG from 5.5 mmol/l to 3.9 mmol/l occurred faster for icodec vs glargine U100 after double dose but not after triple dose (Table 2). There were no statistically significant treatment differences in duration of PG decline from 5.5 mmol/l to 3.0 mmol/l (Table 2).

**Table 2:** Glucodynamics observed following double or triple doses of insulin icodec vs insulin glargine U100

	Double dose				Triple dose			
Endpoint	Insulin icodec	Insulin glargine U100	Treatment ratio (95% CI)^a^	*p* value^b^	Insulin icodec	Insulin glargine U100	Treatment ratio (95% CI)^a^	*p* value^b^
Mean PG_nadir_								
*n*	43	42			38	40		
Estimated geometric mean, mmol/l	3.2	3.3	0.97 (0.94, 1.00)	0.07	3.1	2.9	1.07 (1.04, 1.11)	<0.001
Time to decline from PG_5.5 mmol/l_ to PG_3.9 mmol/l_^c^								
*n*	20	19			20	29		
Estimated geometric mean, h	0.7	1.1	0.62 (0.48, 0.78)	<0.001	0.7	0.7	0.90 (0.78, 1.04)	0.13
Time to decline from PG_5.5 mmol/l_ to PG_3.0 mmol/l_^d^								
*n*	17	15			20	28		
Estimated geometric mean, h	2.9	4.5	0.64 (0.31, 1.33)	0.22	2.4	2.2	1.05 (0.68, 1.64)	0.80
Time to recovery from PG_nadir_ to PG_5.5 mmol/l_^e^								
*n*	15	13			18	22		
Estimated geometric mean, min	28.3	21.0	1.34 (1.16, 1.55)	<0.01	23.8	23.3	1.02 (0.82, 1.28)	0.85
Glucose needed from PG_nadir_ to PG_5.5 mmol/l_^e^								
*n*	15	13			18	22		
Estimated geometric mean, mg/kg	141	111	1.27 (1.05, 1.53)	0.02	116	115	1.02 (0.83, 1.24)	0.88

^a^Insulin icodec vs insulin glargine U100

^b^*p *values derived from two-sided tests of no difference between insulin icodec and insulin glargine U100

^c^Determined in individuals with PG decline to <3.0 mmol/l and/or hypoglycaemia symptoms

^d^Determined in individuals with PG decline to <3.0 mmol/l

^e^Determined in individuals with PG <3.3 mmol/l at the time of initiation of recovery

### Recovery from hypoglycaemia

During recovery from hypoglycaemia at a constant glucose infusion rate of 5.5 mg kg^−1^ min^−1^, it took, on average, slightly less than 30 min and required on average 111–141 mg/kg of glucose to restore PG from PG_nadir_ to 5.5 mmol/l (Table 2). Recovery from PG_nadir_ to PG_5.5 mmol/l_ took slightly longer and required slightly more glucose for icodec vs glargine U100 after double dose but not after triple dose (Table 2).

### Other hypoglycaemic episodes during the treatment periods

During the treatment periods, outside the period of hypoglycaemia induction experiments, there were no severe hypoglycaemic episodes (level 3), and the rate of clinically significant hypoglycaemic episodes (level 2) was comparable for icodec and glargine U100 (3.4 vs 3.0 episodes per participant-year of exposure, respectively). Furthermore, over both doses, the mean±SD duration of level 2 hypoglycaemic episodes was 25.3±8.5 min for icodec and 37.7±15.0 min for glargine U100. There were no level 2 hypoglycaemic episodes during the 48 h prior to any of the hypoglycaemia induction experiments.

### CGM after double and triple doses

CGM data were summarised from the time of double or triple dose until the next insulin administration (excluding the period of hypoglycaemia induction experiments), thereby eliminating any confounding effect of other insulin.

CGM profiles for the first 2 weeks after icodec double dose and for the first week after icodec triple dose are shown in ESM Fig. 2. Mean±SD percentage time spent with glucose <3.0 mmol/l was low across all participants (0.15±0.44% during the first 2 weeks after icodec double dose and 0.29±1.24% during the first week after icodec triple dose) as well as in participants who experienced clinically significant hypoglycaemia during the hypoglycaemia induction experiments (0.21±0.45% during the first 2 weeks after icodec double dose and 0.56±1.70% during the first week after icodec triple dose). The corresponding values for percentage time spent with glucose <3.9 mmol/l were also low at 0.99±2.12% and 1.56±3.54% for all participants, and 1.63±2.75% and 2.67±4.71% for those with clinically significant hypoglycaemia. In accordance with the CGM results, the number of level 2 hypoglycaemic episodes was low from the end of the hypoglycaemia induction experiment until 2 weeks after icodec double dose (four episodes in three individuals) and until 1 week after icodec triple dose (six episodes in five individuals).

CGM profiles for the first 48 h after glargine U100 double and triple doses are shown in ESM Fig. 3. Mean±SD percentage time spent with glucose <3.0 mmol/l was low across all participants (0.01±0.06% after glargine U100 double dose and 0.01±0.06% after glargine U100 triple dose) as well as in those with clinically significant hypoglycaemia during the hypoglycaemia induction experiments (0.00±0.00% and 0.01±0.07%, respectively). The corresponding values for percentage time spent with glucose <3.9 mmol/l were also low at 0.10±0.32% and 0.18±0.46% for all participants, and 0.13±0.37% and 0.23±0.53% for those with clinically significant hypoglycaemia.

### Other safety measures

During the treatment periods, 52 AEs were reported for icodec (10.9 AEs per participant-year of exposure) and 33 AEs were reported for glargine U100 (21.3 AEs per participant-year of exposure). All AEs were mild or moderate, and the majority were assessed by the investigator as being unlikely related to trial product. The most frequently reported AEs were headache, thrombophlebitis, diarrhoea and hypertension. There were no serious AEs or AEs leading to withdrawal. All AEs were recovered at the end of the trial, except for an AE of diabetic foot ulcer, which was assessed as being unlikely related to the trial product.

There were no clinically significant observations for vital signs, electrocardiograms, physical examinations, haematology and biochemistry analyses.

### Analysis of subgroup with clinically significant hypoglycaemia and/or symptoms of hypoglycaemia

**Fig. 4:**
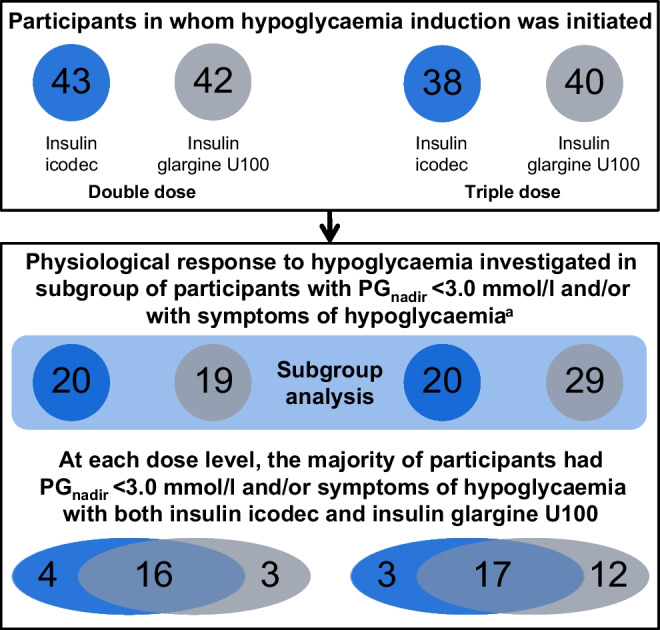
Participant flow diagram showing the number of individuals included in the subgroup analysis of physiological response to hypoglycaemia following double and triple dose of either insulin icodec or insulin glargine U100. ^a^The number of individuals who only had symptoms of hypoglycaemia (i.e. with PG_nadir_ ≥3.0 mmol/l) was *n*=3 for icodec double dose, *n*=4 for glargine U100 double dose, *n*=0 for icodec triple dose and *n*=1 for glargine U100 triple dose

A total of 20/43 (46.5%) and 19/42 (45.2%) participants administered a double dose of icodec or glargine U100, respectively, and 20/38 (52.6%) and 29/40 (72.5%) participants administered a triple dose, respectively, experienced clinically significant hypoglycaemia (PG_nadir_ <3.0 mmol/l) and/or had symptoms of hypoglycaemia as judged by the investigator during hypoglycaemia induction. These participants, therefore, comprised the subgroup for investigation of the physiological response to hypoglycaemia. At each of the two dose levels, there was a high degree of overlap in the participants between the icodec and glargine U100 subgroups, i.e. 16 participants after double dose and 17 participants after triple dose experienced clinically significant hypoglycaemia and/or symptoms of hypoglycaemia after both icodec and glargine U100 (Fig. 4). Baseline characteristics of the subgroup (ESM Table 3) were nominally similar to those in the full group of participants (Table 1) and comparable between icodec and glargine U100 for both double and triple doses.

#### Hypoglycaemia development in the subgroup analysis

Individual PG profiles during hypoglycaemia development in participants with clinically significant hypoglycaemia are shown in ESM Fig. 4. In the subgroup analysis, mean PG_nadir_ was similar for icodec and glargine U100 after double dose (2.9 mmol/l vs 2.9 mmol/l, respectively; treatment ratio 1.01 [95% CI 0.96, 1.07]; *p*=0.58), while it was slightly but statistically significantly higher for icodec vs glargine U100 after triple dose (2.9 mmol/l vs 2.7 mmol/l; treatment ratio 1.05 [95% CI 1.01, 1.10]; *p*=0.02).

#### Hypoglycaemia awareness in the subgroup analysis

After a double dose, the proportion of individuals answering ‘Yes’ to the question ‘Do you feel hypo?’ was nominally higher for icodec vs glargine U100 at PG_3.0_ _mmol/l_ (*n*=9/17 [52.9%] vs *n*=2/15 [13.3%]) and at PG_nadir_ (*n*=14/20 [70.0%] vs *n*=10/19 [52.6%]). After a triple dose, comparable proportions of individuals for icodec vs glargine U100 answered ‘Yes’ to the question ‘Do you feel hypo?’ both at PG_3.0 mmol/l_ (*n*=5/20 [25.0%] vs *n*=8/28 [28.6%]) and at PG_nadir_ (*n*=9/20 [45.0%] vs *n*=15/29 [51.7%]).

#### Hypoglycaemic symptoms in the subgroup analysis

**Fig. 5:**
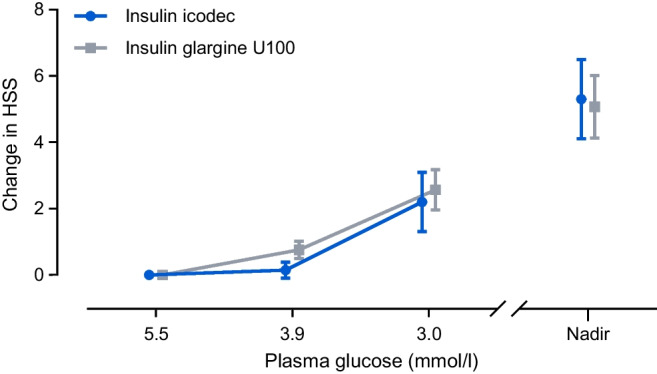
Change from baseline in HSS during development of hypoglycaemia following a triple dose of insulin icodec or insulin glargine U100. Data are mean±SEM. *n*=20 for icodec; *n*=29 for glargine U100, except for PG_3.0_ _mmol/l_ (*n*=28)

Change in HSS from baseline during hypoglycaemia induction is shown in Fig. 5 following a triple dose and in ESM Fig. 5 following a double dose. An increase in HSS was observed during hypoglycaemia induction for both insulin products independent of dose level. There were no statistically significant differences between icodec and glargine U100 (ESM Table 4). Following a triple dose, the mean change in HSS at PG_3.0 mmol/l_ was 2.2 for icodec vs 2.6 for glargine U100 (treatment difference −0.4 [95% CI −2.6, 1.8]; *p*=0.72).

#### Counterregulatory hormones in the subgroup analysis

**Fig. 6:**
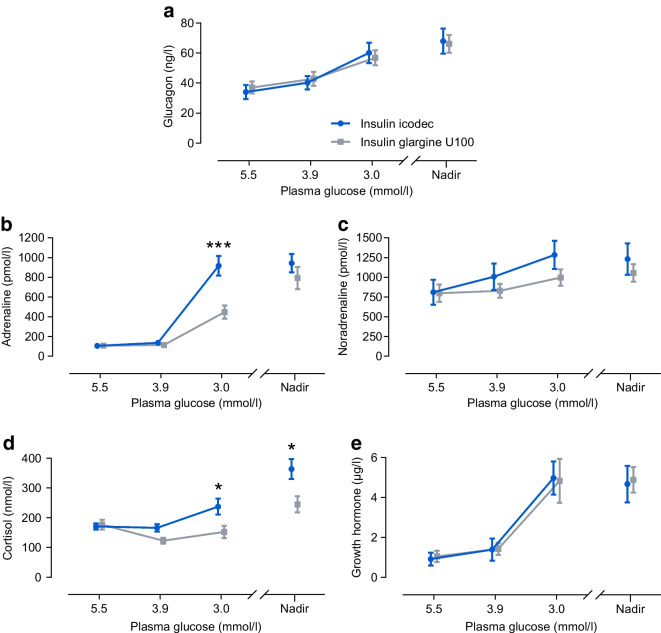
Counterregulatory hormone concentrations during development of hypoglycaemia following a triple dose of insulin icodec or insulin glargine U100. (**a**) Glucagon, (**b**) adrenaline, (**c**) noradrenaline, (**d**) cortisol and (**e**) growth hormone levels are shown. Data are mean±SEM. For (**a**–**c**), *n*=20 for icodec, and *n*=29 for glargine U100 except at PG_3.0_ _mmol/l_ (*n*=28); for (**d**) and (**e**), *n*=20 for icodec except at PG_nadir_ (*n*=19), and *n*=29 for glargine U100 except at PG_3.9_ _mmol/l_ and PG_3.0_ _mmol/l_ (*n*=28). Treatment ratios (95% CI) for icodec/glargine U100 were 2.54 (1.69, 3.82) at PG_3.0_ _mmol/l_ (*p*<0.001) in (**b**), and 1.64 (1.13, 2.38) at PG_3.0_ _mmol/l_ (*p*=0.01) and 1.80 (1.09, 2.97) at PG_nadir_ (*p*=0.02) in (**d**). *p<0.05, ***p<0.001, derived from two-sided tests of no difference between icodec and glargine U100

Concentrations of all five counterregulatory hormones measured appeared to increase from baseline during hypoglycaemia induction for both insulin products following double dose (ESM Fig. 6) and triple dose (Fig. 6). Following a triple dose of insulin, the hormone response was statistically significantly greater with icodec vs glargine U100 for adrenaline at PG_3.0 mmol/l_ (793.8 pmol/l vs 312.2 pmol/l; treatment ratio 2.54 [95% CI 1.69, 3.82]; *p*<0.001), and cortisol at PG_3.0 mmol/l_ (201.4 nmol/l vs 122.9 nmol/l; treatment ratio 1.64 [95% CI 1.13, 2.38]; *p*=0.01) and PG_nadir_ (355.0 nmol/l vs 197.6 nmol/l; treatment ratio 1.80 [95% CI 1.09, 2.97]; *p*=0.02) (ESM Table 5). There were no other statistically significant differences in counterregulatory hormone concentrations between icodec and glargine U100 after double or triple doses (ESM Table 5). Following a triple dose, glucagon concentration at PG_3.0 mmol/l_ was 55.2 ng/l for icodec vs 51.6 ng/l for glargine U100 (treatment ratio 1.07 [95% CI 0.86, 1.33]; *p*=0.53), noradrenaline concentration at PG_3.0 mmol/l_ was 1058.4 pmol/l for icodec vs 843.0 pmol/l for glargine U100 (treatment ratio 1.26 [95% CI 0.89, 1.77]; *p*=0.18) and growth hormone concentration at PG_3.0 mmol/l_ was 3.7 µg/l for icodec vs 2.4 µg/l for glargine U100 (treatment ratio 1.54 [95% CI 0.72, 3.29]; *p*=0.25) (ESM Table 5).

A sensitivity analysis of the five counterregulatory hormones was conducted for both double and triple doses of the insulin products. This sensitivity analysis only included participants who experienced clinically significant hypoglycaemia for both icodec and glargine U100. The sensitivity analysis showed numerical results comparable to the main statistical analysis (data not shown). However, the comparisons of cortisol concentration for icodec vs glargine U100 at PG_3.0_ _mmol/l_ and PG_nadir_ after triple dose did not reach statistical significance in the sensitivity analysis, probably due to the lower number of participants included (data not shown).

#### Vital signs in the subgroup analysis

Diastolic blood pressure, systolic blood pressure and pulse during hypoglycaemia induction are shown in ESM Fig. 7 for both double and triple insulin doses. For both insulin products, diastolic blood pressure appeared to decrease from baseline during hypoglycaemia induction after double and triple doses, while systolic blood pressure was observed to decrease only after double dose. No response was observed in pulse. There were no statistically significant differences in vital signs between icodec and glargine U100 (ESM Table 6).

#### Cognitive function in the subgroup analysis

**Fig. 7:**
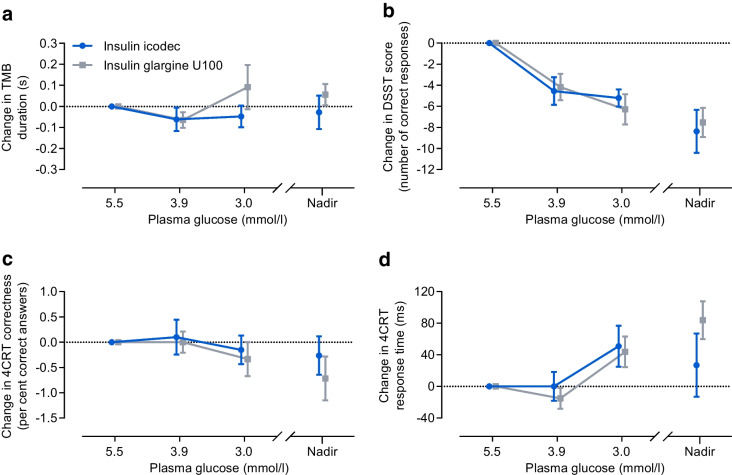
Change from baseline in cognitive function during development of hypoglycaemia following a triple dose of insulin icodec or insulin glargine U100. Changes in (**a**) TMB duration, (**b**) DSST score, (**c**) 4CRT correctness and (**d**) 4CRT response time are shown. Data are mean±SEM. For (**a**), *n*=20 for icodec except at PG_nadir_ (*n*=19), and *n*=29 for glargine U100 except at PG_3.0_ _mmol/l_ (*n*=26) and PG_nadir_ (*n*=28); for (**b**), *n*=20 for icodec except at PG_nadir_ (*n*=19), and *n*=29 for glargine U100 except at PG_3.0_ _mmol/l_ (*n*=28); for (**c**) and (**d**), *n*=20 for icodec except at PG_nadir_ (*n*=19), and *n*=29 for glargine U100 except at PG_3.0_ _mmol/l_ (*n*=27) and PG_nadir_ (*n*=28)

Changes from baseline in cognitive function tests during hypoglycaemia induction are shown in Fig. 7 following a triple dose and in ESM Fig. 8 following a double dose of the insulin products. A nominal decrease in number of correct responses in the DSST and increase in response time in the 4CRT test were observed during development of hypoglycaemia with both icodec and glargine U100 independent of dose level. There were no major changes observed in TMB duration or in the percentage of correct answers in the 4CRT test. There were no statistically significant differences between icodec and glargine U100 (ESM Table 7).

#### Counterregulatory hormones and vital signs during recovery from hypoglycaemia in the subgroup analysis

Counterregulatory hormone concentrations and vital signs during recovery from hypoglycaemia following the double or triple doses of insulin are shown in ESM Figs 9–11. As PG returned to normal levels, counterregulatory hormone concentrations, diastolic and systolic blood pressure and pulse generally returned towards baseline for both insulin products. There were no apparent differences between icodec and glargine U100.

## Discussion

In the current study, the main findings related to hypoglycaemia frequency and glucodynamics were that the risk of hypoglycaemia was comparable for icodec vs glargine U100 following double and triple doses, the duration of decline in PG towards clinically significant hypoglycaemia (PG_3.0 mmol/l_) was also comparable for both insulin products, and full recovery from hypoglycaemia was achieved within 30 min during constant i.v. glucose infusion at 5.5 mg kg^−1^ min^−1^ for both icodec and glargine U100. The main finding related to hypoglycaemia counterregulation was that there was a robust symptomatic and endocrine response in participants with clinically significant hypoglycaemia, i.e. with PG_nadir_ <3.0 mmol/l and/or symptoms of hypoglycaemia. Overall, HSS and counterregulatory hormone levels increased to the same extent for icodec vs glargine U100. The only exception was a moderately greater increase in adrenaline and cortisol in response to hypoglycaemia following a triple dose of icodec.

Phase 2 clinical trials up to 26 weeks in insulin-naive or previously insulin-treated individuals with type 2 diabetes have shown low rates of level 2 and level 3 hypoglycaemia for once-weekly icodec that are comparable to once-daily glargine U100 [11–13]. The results from the current study support these Phase 2 trial findings and can be further extended to suggest that, even in situations when there is a substantial mismatch between the insulin dose administered and the amount of insulin required, once-weekly icodec is not associated with an increased risk of hypoglycaemia compared with once-daily glargine U100.

Avoiding hypoglycaemia is obviously a focus point for individuals with diabetes [1–3]. However, in the unfortunate situation that a hypoglycaemic episode occurs, it is just as important to be able to rapidly correct the fall in PG. Given the longer dosing interval and the longer duration of action of once-weekly insulin, prolonged hypoglycaemia, slow recovery from hypoglycaemia and recurrent hypoglycaemia might be a concern. However, results from the current trial and from Phase 2 trials with icodec are reassuring. Thus, a post hoc analysis based on CGM data from two Phase 2 trials showed similar duration of hypoglycaemic episodes with icodec vs glargine U100 in type 2 diabetes (R. J. Silver, unpublished results). In the current trial, recovery from hypoglycaemia by constant i.v. glucose infusion at 5.5 mg kg^−1^ min^−1^ took <30 min for all treatments (Table 2). After the hypoglycaemia induction experiment until 2 weeks after the double icodec dose, until 1 week after the triple icodec dose and until 48 h after the glargine U100 double and triple doses (i.e. the longest possible assessment period with no other potentially confounding insulin administration), the mean percentage time spent below range based on CGM recordings was well below the consensus guidance clinical targets for CGM data of <1% of time spent with glucose <3.0 mmol/l and <4% of time spent with glucose <3.9 mmol/l [20, 21]. This was the case both in all participants and in the subgroup of individuals who experienced clinically significant hypoglycaemia during the hypoglycaemia induction experiments. Furthermore, no severe (level 3) and very few PG-confirmed (level 2) hypoglycaemic episodes were recorded following the hypoglycaemia induction experiment when assessed up to 2 weeks after icodec double dose and 1 week after icodec triple dose. It must, however, be taken into consideration that participants and site staff were specifically focused on preventing hypoglycaemia in the periods after the hypoglycaemia experiments. In addition, it must be acknowledged that the 48 h period of CGM data for glargine U100 is substantially shorter than the recommended minimum 14 days [21]. Finally, as the next dose of insulin after the double or triple dose was skipped in the current trial, it reflects the clinical situation when administration of a higher-than-normal dose is discovered and mitigated prior to the next planned dose. In the scenario where a higher-than-normal dose is not realised, insulin exposure will remain slightly elevated until the normal steady-state level is re-established, expectedly within 3–4 half lives of the insulin product. Importantly, however, insulin exposure and, consequently, the risk of hypoglycaemia are greatest during the first dosing interval after a higher-than-normal dose.

The present findings may not only apply to situations where an unintentionally high insulin dose is administered, e.g. due to miscalculation of the insulin dose or forgetting that a dose has been taken and hence taking two weekly doses close to each other. Rather, they may also be generalised to situations where the usually administered insulin dose is too high to match the insulin needs, due to reduced insulin requirement. This could occur during and after exercise, during intercurrent illness, or during hospitalisation that often implies fasting prior to medical testing or operative procedures [7]. Such situations often represent increased risk of developing hypoglycaemia in individuals with diabetes [22, 23]. Double and triple doses of once-weekly icodec are equivalent to 14 and 21 times the normal daily insulin dose or to 2 and 3 weeks of normal weekly insulin doses, respectively. Despite this vast amount of icodec administered, it is reassuring to see in the current study that when the next weekly dose was omitted, the risk of hypoglycaemia was not higher in comparison with double or triple doses of once-daily glargine U100 followed by omission of the next daily dose. Furthermore, recovery from hypoglycaemia did not require more glucose for icodec vs glargine U100 after triple dose and only slightly more glucose for icodec vs glargine U100 after double dose.

The considerably extended half-life of icodec is mainly due to strong, reversible binding to albumin, whereby an essentially inactive depot of icodec is formed throughout the circulation and the interstitial compartment, from which icodec is slowly and continuously released [9, 10]. In addition, the low insulin receptor binding affinity of icodec contributes to its long half-life by reducing the clearance rate, as insulin is mainly cleared by internalisation following binding to and activation of the insulin receptor [9, 10]. In case of excess amounts of icodec throughout the body, e.g. when there is an acute reduction in the need for insulin, the essentially inactive albumin-bound depot of icodec serves as a buffer to prevent a rapid increase in the active pool of icodec that is readily available to the insulin receptor. Moreover, the low insulin receptor binding affinity of icodec prevents an extensively inappropriate glucose-lowering effect [10]. In addition, in individuals with type 2 diabetes, it appears generally difficult to induce clinically significant hypoglycaemia even when administering double or triple the usual dose of icodec (Fig. 3). This is in contrast to previous hypoglycaemia induction studies in type 1 diabetes, where close-to-normal doses of insulin detemir and neutral protamine Hagedorn insulin led to clinically significant hypoglycaemia in 78% of participants and triple doses of insulin degludec and glargine U100 led to clinically significant hypoglycaemia in almost all participants [8, 24]. Once-daily insulin degludec is another albumin-bound insulin and was shown in double-blind crossover Phase 3 trials to be associated with a reduced rate of overall symptomatic hypoglycaemia during 32 weeks of treatment as compared with once-daily glargine U100 in both type 1 and type 2 diabetes [25, 26]. It may be hypothesised that albumin-binding as a protraction mechanism has beneficial effects in reducing the risk of hypoglycaemia, which would then also favourably impact the hypoglycaemia profile of once-weekly icodec.

Previously, the symptomatic and endocrine responses to hypoglycaemia have been studied for insulin detemir, insulin degludec and glargine U100 in healthy individuals and in individuals with type 1 diabetes [8, 24, 27–29]. Furthermore, several studies have investigated the response to hypoglycaemia induced by human soluble insulin under hyperinsulinaemic–hypoglycaemic clamp conditions in individuals with type 2 diabetes [30]. However, to our knowledge, this is the first study to investigate the physiological response to hypoglycaemia induced by a basal insulin analogue in individuals with type 2 diabetes. It was reassuring that the physiological response to hypoglycaemia was as large for icodec as for the widely used basal insulin glargine U100. These findings suggest that when used in clinical practice, icodec is likely to induce a similar counterregulatory response to imminent and/or actual hypoglycaemia compared with glargine U100. Furthermore, hypoglycaemia awareness and counterregulation are particularly important in preventing severe hypoglycaemia [31, 32]. Thus, the current findings are in accordance with the results from Phase 2 clinical trials, which show comparably low rates of level 2 (blood glucose <3.0 mmol/l) and level 3 (severe) hypoglycaemia [11–13] as well as similar duration of hypoglycaemic episodes (R. J. Silver, unpublished results) for once-weekly icodec vs once-daily glargine U100.

Increased secretion of adrenaline from the adrenal medulla during hypoglycaemia development plays a role in early counterregulation by increasing endogenous glucose production and reducing insulin-stimulated glucose uptake [33–35]. In the current study, a greater adrenaline response was seen after triple dose for icodec vs glargine U100 at PG_3.0 mmol/l_ but not at PG_nadir_. This indicates that adrenaline secretion was triggered at a higher PG threshold for icodec vs glargine U100, which may potentially explain why numerically fewer participants experienced clinically significant hypoglycaemia after triple dose for icodec vs glargine U100 and why PG declined to ≤2.5 mmol/l in statistically significantly fewer participants for icodec vs glargine U100 (Fig. 3). Increased cortisol secretion from the adrenal cortex helps to prevent a substantial PG decline during prolonged hypoglycaemia by increasing glucose production and inhibiting glucose disposal [34, 35]. Thus, the greater cortisol response at both PG_3.0_ _mmol/l_ and PG_nadir_ after triple dose for icodec vs glargine U100 could explain why PG did not ultimately fall as low with icodec vs glargine U100 after triple dose (PG_nadir_ of 3.1 mmol/l vs 2.9 mmol/l, respectively; *p*<0.001). Interestingly, in a trial in type 1 diabetes investigating the acute physiological response to hypoglycaemia, growth hormone and cortisol responses were statistically significantly greater with insulin degludec vs glargine U100, while the observed higher adrenaline response with insulin degludec vs glargine U100 did not reach statistical significance [8]. While chronically elevated adrenaline and cortisol may have broad deleterious implications, the beneficial impact of acute increases in these stress hormones, e.g. during hypoglycaemia, is well established [33–37].

In individuals with type 2 diabetes who are recently diagnosed, and in those where the disease has not yet progressed to the level where the glucagon secretory capacity becomes absent, glucagon is known to be the most important counterregulatory hormone [34, 38]. In the current study, in individuals with at least some remaining glucagon secretion, glucagon increased similarly in response to hypoglycaemia with icodec and glargine U100. The similar glucagon response might explain why the slight treatment differences in adrenaline and cortisol responses after triple dose did not translate into differences in other clinical signs such as HSS, cognitive function or vital signs.

There is no apparent explanation why adrenaline and cortisol responses were greater with icodec vs glargine U100 after triple dose. A potential reason might be impaired physiological response to hypoglycaemia caused by antecedent hypoglycaemia due to the shorter period of 7 days between the two hypoglycaemia inductions during glargine U100 treatment in contrast to the 3 weeks between hypoglycaemia inductions during icodec treatment. However, the effect of antecedent hypoglycaemia has been shown to last less than 2 days [39]. Another potential reason could be related to the open-label trial design. When participants knowingly received the novel once-weekly basal insulin icodec, they may have become slightly distressed, hence triggering the moderately elevated adrenergic hormone response. However, it would then still be difficult to explain why this did not occur after the double dose of icodec.

A strength of the trial was the enrolment of individuals with type 2 diabetes, the population which could potentially benefit the most from once-weekly basal insulin. At the same time, it must be recognised that the current results do not necessarily apply to individuals with type 1 diabetes. To mimic a clinically relevant situation, hypoglycaemia induction was carried out following a high dose given on top of the individually titrated normal dose at steady state and using the intended dosing frequency of once per week for icodec. Furthermore, s.c. administration was used in contrast to many previous studies using an i.v. hyperinsulinaemic–hypoglycaemic glucose clamp to study hypoglycaemia under controlled conditions [30]. The threshold of 3.0 mmol/l for clinically significant hypoglycaemia was chosen according to common consensus [40]. However, it should be kept in mind that the current hypoglycaemia inductions were carried out in a strictly controlled experimental setting and may not entirely reflect spontaneous hypoglycaemic episodes induced in clinical practice by two insulin products with very different pharmacodynamic properties. The aim was to induce hypoglycaemia at the time of maximum glucose-lowering effect for both insulin products to facilitate appropriate comparison. However, minor variation in time to maximum glucose-lowering effect may have had a slight impact on the relative rate and extent of glucose decline, as well as the time and amount of glucose required to recover from hypoglycaemia for icodec vs glargine U100, as specifically seen after double dose in the current trial.

The subgroup approach used to investigate the physiological response to hypoglycaemia, whereby participants were only included in the analysis if they actually experienced hypoglycaemia during the hypoglycaemic induction experiment, could potentially have led to selection bias. However, baseline characteristics in the subgroup were similar to those in the full group of participants, thus reducing the likelihood of such bias. Furthermore, not all participants contributed to hypoglycaemia induction results for both icodec and glargine U100, which might have also had an impact on the conclusions. However, in a sensitivity analysis of the counterregulatory hormone responses that only included individuals who experienced hypoglycaemia for both insulin products, numerical results comparable to the main statistical analyses were obtained. It cannot be excluded that the substantial number of statistical comparisons performed in the trial with no multiplicity adjustment may have led to type 1 error(s). On the contrary, the lack of treatment differences in cognitive function tests, despite greater responses in adrenaline and cortisol, with icodec vs glargine U100 after triple dose could have been due to the relatively small sample size combined with the inherently large variation in the cognitive function measures.

In conclusion, it is reassuring that double and triple doses of once-weekly icodec do not lead to an increased risk of hypoglycaemia compared with once-daily glargine U100, and that the time to develop hypoglycaemia and recover from hypoglycaemia are comparable for icodec and glargine U100. The study also suggests that hypoglycaemia induced by icodec is accompanied by symptomatic and counterregulatory responses, at least as robust as those seen for glargine U100, to support restoration of euglycaemia. Thus, the current study provides further reassurance about the safety of once-weekly icodec.

## Supplementary Information

Below is the link to the electronic supplementary material.

Supplementary file1 (PDF 3326 KB)

## Data Availability

Data will be shared with bona fide researchers submitting a research proposal requesting access to data for use as approved by the Independent Review Board according to the Independent Review Board Charter (see novonordisk-trials.com). The data will be made available on a specialised SAS data platform. Individual participant data will be shared in data sets in a de-identified/anonymised format. Data sets from Novo Nordisk sponsored clinical research completed after 2001 for product indications approved in both the EU and USA will be shared. Study protocol and redacted Clinical Study Report will be available according to Novo Nordisk data sharing commitments. The data will be available permanently after research completion and approval of product and product use in both the EU and USA. There is no end date.

## Abbreviations

AE: Adverse event
AUC_τ_: AUC during one dosing interval
CGM: Continuous glucose monitoring
C_max_: Maximum concentration
4CRT: Four-choice reaction time
DSST: Digit symbol substitution test
HSS: Hypoglycaemic symptoms score
PG: Plasma glucose
TMB: Trail making B

## Authors’ relationships and activities

TRP has received research support (directed to Medical University of Graz, Graz, Austria) from AstraZeneca, Novo Nordisk and Sanofi, has served as speaker, board member and in advisory panels for Arecor and Novo Nordisk and is an employee of CBmed – Center for Biomarker Research in Medicine. KNA, RC, SK and HH are employees and shareholders of Novo Nordisk. ES has received travel grants from Mylan. All other authors declare that there are no relationships or activities that might bias, or be perceived to bias, their work.
