# Supplementary material for: Hypoglycaemia frequency and physiological response after double or triple doses of once-weekly insulin icodec vs once-daily insulin glargine U100 in type 2 diabetes: a randomised crossover trial
Source: Diabetologia. 2023 Jun 13;66(8):1413–30. doi: 10.1007/s00125-023-05921-8 (PMC10317887; doi:10.1007/s00125-023-05921-8)
Supplement: Supplementary file 1 — Supplementary file1 (PDF 3326 KB) [file 125_2023_5921_MOESM1_ESM.pdf]

Hypoglycaemia frequency and physiological response after double or triple doses of once-weekly insulin icodec vs once-daily insulin glargine U100 in type 2 diabetes: a randomised crossover trial

Thomas R. Pieber, Kristine N. Arfelt, Roman Cailleteau, Marlies Hart, Soumitra Kar, Ines Mursic, Eva Svehlikova, Martina Urschitz, Hanne Haahr

Electronic supplementary material

ESM Methods

Procedures and assessments The following describes handling of scenarios with PG_{nadir} >2.5 mmol/l, slow PG decline towards hypoglycaemia and/or slow recovery from hypoglycaemia.

PG_{nadir} >2.5 mmol/l with unacceptable hypoglycaemic symptoms

If possible, the PG_{nadir} level was maintained for 15 min to allow for hypoglycaemic response assessments, and procedures for recovery from hypoglycaemia were performed as planned.

2.5 mmol/l < PG_{nadir} < 3.0 mmol/l with or without hypoglycaemic symptoms – and no further decline in PG expected

PG_{nadir} was maintained for 15 min and procedures for recovery from hypoglycaemia were performed as planned.

PG_{nadir} ≥3.0 mmol/l with hypoglycaemic symptoms – and no further decline in PG expected

PG_{nadir} was maintained for 15 min and procedures for recovery from hypoglycaemia were performed as planned.

PG_{nadir} ≥3.0 mmol/l without hypoglycaemic symptoms

If assessed to be safe, the hypoglycaemia induction was continued until 07:00 hours followed by restorage of euglycaemia. The constant i.v. glucose infusion (5.5 mg kg^{-1} min^{-1}) following PG_{nadir} was not performed. The scheduled hypoglycaemic response assessments corresponding to $PG_{3.0\ mmol/l}$ and PG_{nadir}

during hypoglycaemia development and $PG_{3.0\ mmol/l}$, $PG_{3.9\ mmol/l}$ and $PG_{5.5\ mmol/l}$ during recovery from hypoglycaemia were also not performed.

During recovery from hypoglycaemia, euglycaemia ($PG_{5.5\ mmol/l}$) was not restored by 08:30 hours

The constant i.v. glucose infusion (5.5 mg kg^{-1} min^{-1}) was continued until euglycaemia was restored, followed by maintenance of euglycaemia by a variable i.v. glucose infusion for a minimum of 30 min.

Pharmacokinetic modeling For insulin icodec, the structural part of the pharmacokinetic model was a one-compartment model with first-order absorption and first-order elimination, parameterised in terms of an absorption rate parameter (k_A), a clearance parameter (CL/F) and a volume of distribution parameter (V/F). Inter-individual variability was included on CL/F and V/F, assuming a log-normal distribution with correlation between the two parameters. Between-occasion (between-dosing interval) variability was included on k_A and on the bioavailability parameter (F), which was set to 1 on average. A combined (proportional + additive) error model was used to describe residual variability.

For insulin glargine, the structural part of the pharmacokinetic model was a one-compartment model with first-order absorption through a single transit compartment and first-order elimination. In addition, the model assumed a split of the dose between the depot and transit compartments to reflect fast absorption of a small fraction of the dose. The model was parameterised in terms of a fast fraction parameter (F_{fast}), a slow absorption rate parameter ($k_{A,slow}$) from the depot compartment to the transit compartment, a fast absorption rate parameter ($k_{A,fast}$) from the transit compartment to the central compartment, a clearance parameter (CL/F) and a volume of distribution parameter (V/F). Inter-individual variability was included on F_{fast}, $k_{A,slow}$ and CL/F, assuming a log-normal distribution with no correlation. Between-occasion (between-dosing interval) variability was included on $k_{A,slow}$ and on the bioavailability parameter (F), which was set to 1 on average.
A combined (proportional + additive) error model was used to describe residual variability.

ESM Table 1 Blood sampling for pharmacokinetic analysis of insulin icodec

Day in treatment period	Nominal time[a] (h)	Insulin icodec
1	00[b]	X
8	00[b]	X
9	12	X
10	48	X
15	00[b]	X
16	06	X
	12	X
	18	X
	24	X
17	30	X
	36	X
	48	X
18	60	X
	72	X
22	168	X
29	00[b]	X
36	00[b]	X
37	06	X
	12	X
	18	X
	24	X
38	30	X
	36	X
	48	X
39	60	X
	72	X
43	168	X

[a]Relative to previous icodec dose administered at 20:00 hours
[b]Pre-dose

ESM Table 2 Blood sampling for pharmacokinetic analysis of insulin glargine

Day in treatment period	Nominal time[a] (h)	Insulin glargine
3	00[b]	X
	02	X
	04	X
	06	X
	08	X
	10	X
	12	X
4	16	X
	20	X
	00[b]	X
	02	X
	04	X
	06	X
	08	X
	10	X
	12	X
5	16	X
	20	X
	24	X
10	00[b]	X
11	00[b]	X
	02	X
	04	X
	06	X
	08	X
	10	X
	12	X
12	16	X
	20	X
	24	X

[a]Relative to previous glargine U100 dose administered at 09:00 hours
[b]Pre-dose

ESM Table 3 Baseline characteristics for the subgroup analysis of physiological response to hypoglycaemia, i.e. in individuals with PG_{nadir} <3.0 mmol/l and/or symptoms of hypoglycaemia following double or triple doses of insulin icodec or insulin glargine U100

	Double dose		Triple dose	
	Insulin icodec	Insulin glargine U100	Insulin icodec	Insulin glargine U100
N	20	19	20	29
Age, years	56.6±10.1	57.9±8.6	59.6±6.7	56.5±9.9
Sex				
Men	10 (50.0)	12 (63.2)	15 (75.0)	19 (65.5)
Women	10 (50.0)	7 (36.8)	5 (25.0)	10 (34.5)
Race				
White	20 (100.0)	18 (94.7)	20 (100.0)	28 (96.6)
Asian Indian	0 (0.0)	1 (5.3)	0 (0.0)	1 (3.4)
Body weight, kg	82.9±12.8	83.7±13.4	85.4±12.7	85.3±13.8
Height, m	1.72±0.09	1.71±0.09	1.73±0.10	1.74±0.10
BMI, kg/m^2	28.1±4.1	28.6±4.2	28.6±4.5	28.2±4.0
HbA_{1c}, mmol/mol	56±9	57±8	57±7	56±7
HbA_{1c}, %	7.3±0.8	7.4±0.7	7.4±0.7	7.2±0.7
Fasting PG, mmol/l	7.2±1.8	7.3±1.9	7.4±1.7	7.2±1.6
Fasting C-peptide, nmol/l	0.6±0.3	0.7±0.8	0.6±0.3	0.7±0.7
Diabetes duration, years	13.9±8.9	14.8±8.6	15.0±8.5	14.8±8.6
Any oral glucose-lowering drug at screening	19 (95.0)	18 (94.7)	19 (95.0)	27 (93.1)
Individual once-daily basal insulin dose, U/kg	0.41±0.18	0.39±0.14	0.43±0.17	0.36±0.14

Data are mean±SD or *n* (%)

ESM Table 4 Comparison of change from baseline in HSS during hypoglycaemia following double or triple doses of insulin icodec vs insulin glargine U100

Endpoint	Double dose		Triple dose	
	Insulin icodec	Insulin glargine U100	Insulin icodec	Insulin glargine U100
**Change in HSS at $PG_{3.0\ mmol/l}$**				
n	17	15	20	28
Least-squares mean	1.8	0.6	2.2	2.6
Treatment difference (95% CI)[a]	1.2 (-1.5, 4.0)		-0.4 (-2.6, 1.8)	
p value[b]	0.36		0.72	
**Change in HSS at PG_{nadir}**				
n	20	19	20	29
Least-squares mean	6.0	6.3	5.4	5.0
Treatment difference (95% CI)[a]	-0.3 (-4.0, 3.5)		0.5 (-2.7, 3.6)	
p value[b]	0.88		0.77	

[a]Icodec vs glargine U100
[b]*p* values derived from two-sided tests of no difference between icodec and glargine U100

ESM Table 5 Comparison of counterregulatory hormone concentrations during hypoglycaemia following double or triple doses of insulin icodec vs insulin glargine U100

Endpoint	Double dose		Triple dose	
	Insulin icodec	Insulin glargine U100	Insulin icodec	Insulin glargine U100
**Glucagon at $PG_{3.0\ mmol/l}$**				
n	17	15	20	28
Estimated geometric mean, ng/l	39.6	47.7	55.2	51.6
Treatment ratio (95% CI)[a]	0.83 (0.59, 1.16)		1.07 (0.86, 1.33)	
p value[b]	0.24		0.53	
**Glucagon at PG_{nadir}**				
n	19	19	20	29
Estimated geometric mean, ng/l	48.9	53.1	59.8	59.1
Treatment ratio (95% CI)[a]	0.92 (0.69, 1.23)		1.01 (0.74, 1.38)	
p value[b]	0.54		0.93	
**Adrenaline at $PG_{3.0\ mmol/l}$**				
n	17	15	20	28
Estimated geometric mean, pmol/l	476.7	528.3	793.8	312.2
Treatment ratio (95% CI)[a]	0.90 (0.54, 1.52)		2.54 (1.69, 3.82)	
p value[b]	0.66		<0.001	
**Adrenaline at PG_{nadir}**				
n	20	19	20	29
Estimated geometric mean, pmol/l	530.4	590.9	735.8	570.9
Treatment ratio (95% CI)[a]	0.90 (0.62, 1.30)		1.29 (0.88, 1.89)	
p value[b]	0.54		0.18	
**Noradrenaline at $PG_{3.0\ mmol/l}$**				
n	17	15	20	28
Estimated geometric mean, pmol/l	952.7	853.0	1058.4	843.0
Treatment ratio (95% CI)[a]	1.12 (0.83, 1.50)		1.26 (0.89, 1.77)	
p value[b]	0.42		0.18	
**Noradrenaline at PG_{nadir}**				
n	20	19	20	29
Estimated geometric mean, pmol/l	1078.0	907.4	934.1	890.5
Treatment ratio (95% CI)[a]	1.19 (0.91, 1.55)		1.05 (0.79, 1.40)	
p value[b]	0.19		0.73	
**Cortisol at $PG_{3.0\ mmol/l}$**				
n	17	15	20	28
Estimated geometric mean, nmol/l	245.9	153.8	201.4	122.9
Treatment ratio (95% CI)[a]	1.60 (0.91, 2.80)		1.64 (1.13, 2.38)	
p value[b]	0.09		0.01	

Endpoint	Double dose		Triple dose	
	Insulin icodec	Insulin glargine U100	Insulin icodec	Insulin glargine U100
**Cortisol at PG_{nadir}**				
n	19	19	19	29
Estimated geometric mean, nmol/l	296.0	300.3	355.0	197.6
Treatment ratio (95% CI)[a]	0.99 (0.67, 1.45)		1.80 (1.09, 2.97)	
p value[b]	0.93		0.02	
**Growth hormone at $PG_{3.0\ mmol/l}$**				
n	17	15	20	28
Estimated geometric mean, µg/l	3.6	4.1	3.7	2.4
Treatment ratio (95% CI)[a]	0.89 (0.46, 1.75)		1.54 (0.72, 3.29)	
p value[b]	0.72		0.25	
**Growth hormone at PG_{nadir}**				
n	19	19	19	29
Estimated geometric mean, µg/l	4.7	3.5	3.8	3.5
Treatment ratio (95% CI)[a]	1.35 (0.94, 1.94)		1.10 (0.50, 2.43)	
p value[b]	0.10		0.80	

[a]Icodec vs glargine U100
[b]*p* values derived from two-sided tests of no difference between icodec and glargine U100

ESM Table 6 Comparison of vital signs during hypoglycaemia following double or triple doses of insulin icodec vs insulin glargine U100

Endpoint	Double dose		Triple dose	
	Insulin icodec	Insulin glargine U100	Insulin icodec	Insulin glargine U100
**Change in DBP at $PG_{3.0\ mmol/l}$**				
n	17	15	20	28
Least-squares mean, mmHg	-6.1	-7.7	-2.4	-3.2
Treatment difference (95% CI)[a]	1.7 (-3.1, 6.4)		0.8 (-3.4, 5.0)	
p value[b]	0.47		0.69	
**Change in DBP at PG_{nadir}**				
n	20	19	20	29
Least-squares mean, mmHg	-5.4	-5.9	-1.1	-4.7
Treatment difference (95% CI)[a]	0.5 (-4.0, 4.9)		3.6 (-0.8, 8.0)	
p value[b]	0.82		0.10	
**Change in SBP at $PG_{3.0\ mmol/l}$**				
n	17	15	20	28
Least-squares mean, mmHg	-4.9	-8.6	-0.9	-1.6
Treatment difference (95% CI)[a]	3.7 (-4.3, 11.7)		0.7 (-6.6, 8.0)	
p value[b]	0.33		0.84	
**Change in SBP at PG_{nadir}**				
n	20	19	20	29
Least-squares mean, mmHg	-7.7	-2.5	1.8	-2.1
Treatment difference (95% CI)[a]	-5.2 (-16.5, 6.1)		3.9 (-2.2, 10.0)	
p value[b]	0.33		0.19	
**Change in pulse at $PG_{3.0\ mmol/l}$**				
n	17	15	20	28
Least-squares mean, beats/min	-1.3	-0.5	3.1	2.4
Treatment difference (95% CI)[a]	-0.9 (-7.4, 5.7)		0.8 (-4.4, 5.9)	
p value[b]	0.77		0.75	
**Change in pulse at PG_{nadir}**				
n	20	19	20	29
Least-squares mean, beats/min	1.2	1.0	2.8	2.3
Treatment difference (95% CI)[a]	0.2 (-5.0, 5.4)		0.5 (-4.8, 5.7)	
p value[b]	0.94		0.86	

[a]Icodec vs glargine U100
[b]*p* values derived from two-sided tests of no difference between icodec and glargine U100
DBP, diastolic blood pressure; SBP, systolic blood pressure

ESM Table 7 Comparison of cognitive function during hypoglycaemia following double or triple doses of insulin icodec vs insulin glargine U100

Endpoint	Double dose		Triple dose	
	Insulin icodec	Insulin glargine U100	Insulin icodec	Insulin glargine U100
**Change in TMB at $PG_{3.0\ mmol/l}$**				
n	16	15	20	26
Least-squares mean, sec	0.3	-0.1	-0.0	0.1
Treatment difference (95% CI)[a]	0.4 (-0.3, 1.0)		-0.1 (-0.4, 0.2)	
p value[b]	0.22		0.58	
**Change in TMB at PG_{nadir}**				
n	18	18	19	28
Least-squares mean, sec	0.3	-0.0	-0.0	0.1
Treatment difference (95% CI)[a]	0.3 (-0.2, 0.9)		-0.1 (-0.3, 0.1)	
p value[b]	0.23		0.43	
**Change in DSST at $PG_{3.0\ mmol/l}$**				
n	16	15	20	28
Least-squares mean, number of correct responses	-5.4	-6.6	-5.5	-6.0
Treatment difference (95% CI)[a]	1.2 (-3.1, 5.5)		0.5 (-3.3, 4.3)	
p value[b]	0.57		0.78	
**Change in DSST at PG_{nadir}**				
n	18	18	19	29
Least-squares mean, number of correct responses	-6.7	-7.3	-9.1	-7.0
Treatment difference (95% CI)[a]	0.6 (-3.7, 4.9)		-2.2 (-6.8, 2.5)	
p value[b]	0.77		0.34	
**Change in 4CRT correctness at $PG_{3.0\ mmol/l}$**				
n	16	15	20	27
Least-squares mean, %-points	0.1	-0.1	-0.3	-0.4
Treatment difference (95% CI)[a]	0.2 (-1.1, 1.4)		0.1 (-0.7, 0.9)	
p value[b]	0.75		0.78	
**Change in 4CRT correctness at PG_{nadir}**				
n	18	17	19	28
Least-squares mean, %-points	-0.3	-0.1	-0.3	-0.7
Treatment difference (95% CI)[a]	-0.2 (-1.3, 0.9)		0.4 (-0.9, 1.7)	
p value[b]	0.75		0.55	
**Change in 4CRT response time at $PG_{3.0\ mmol/l}$**				
n	16	15	20	27
Least-squares mean, msec	19.7	99.5	55.4	43.6
Treatment difference (95% CI)[a]	-79.7 (-170.4, 10.9)		11.8 (-44.0, 67.6)	
p value[b]	0.08		0.66	

Endpoint	Double dose		Triple dose	
	Insulin icodec	Insulin glargine U100	Insulin icodec	Insulin glargine U100
**Change in 4CRT response time at PG_{nadir}**				
n	18	17	19	28
Least-squares mean, msec	40.4	137.4	21.3	88.0
Treatment difference (95% CI)[a]	-97.1 (-230.5, 36.3)		-66.7 (-155.8, 22.4)	
p value[b]	0.15		0.14	

[a]Icodec vs glargine U100
[b]*p* values derived from two-sided tests of no difference between icodec and glargine U100

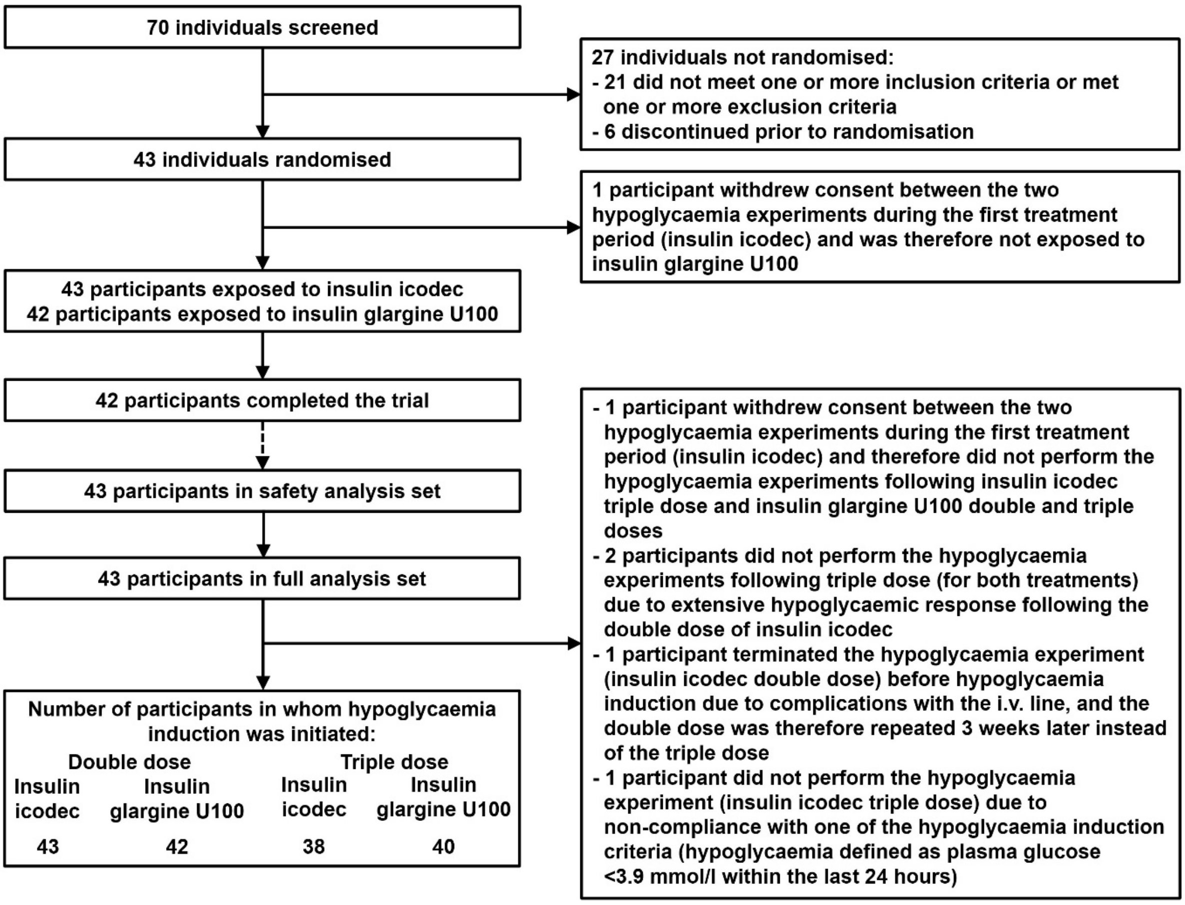

ESM Fig. 1 Participant disposition

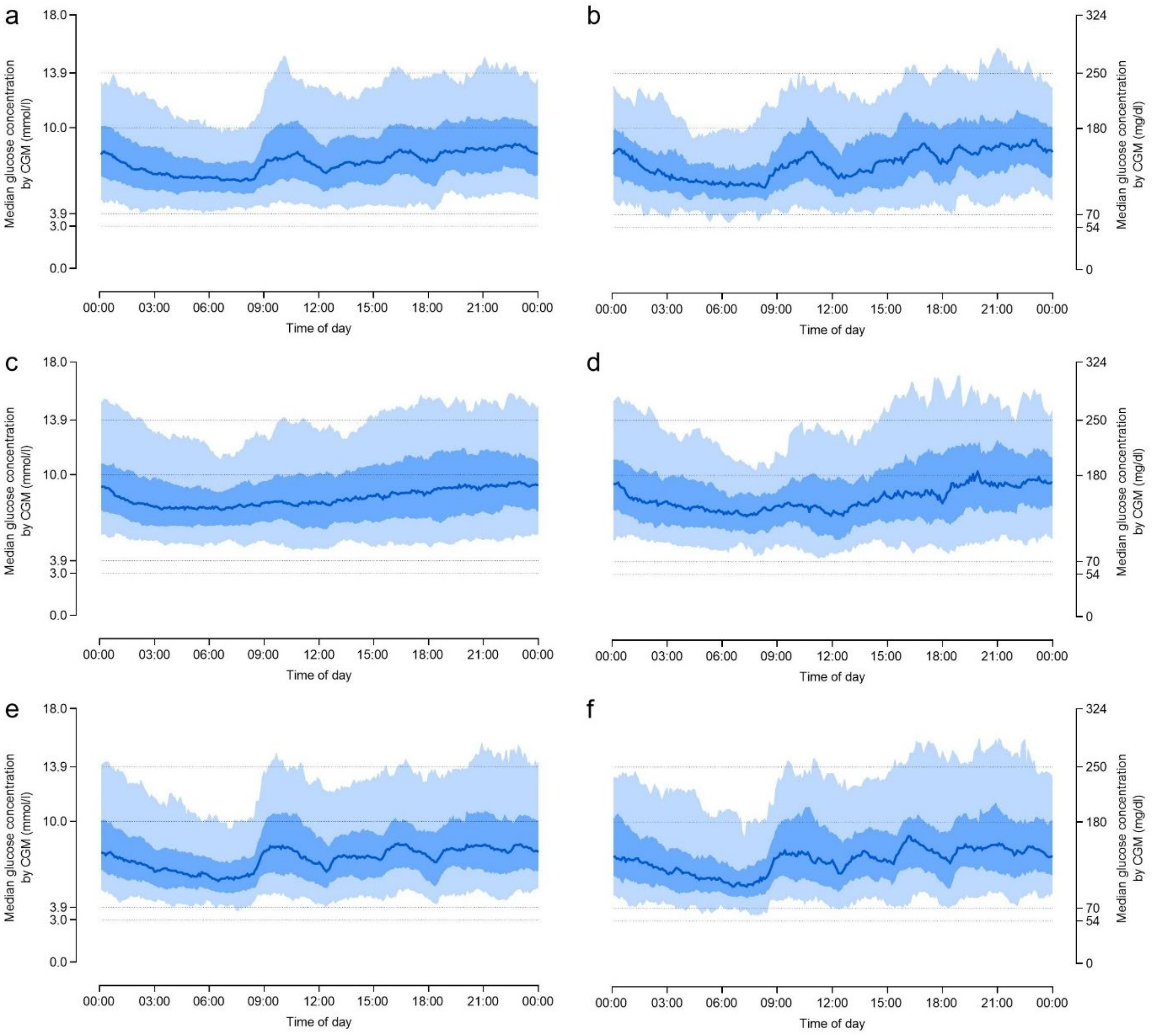

ESM Fig. 2 Median daily CGM profiles following a double or triple dose of insulin icodec in all participants (**a**, **c**, **e**) and in participants with PG_{nadir} <3.0 mmol/l during hypoglycaemia induction (**b**, **d**, **f**)

(**a**, **b**) First week after double dose, (**c**, **d**) second week after double dose and (**e**, **f**) first week after triple dose are shown. Curves are median values. Dark blue bands represent the 25th to 75th percentiles and light blue bands represent the 5th to 95th percentiles. CGM data obtained during hypoglycaemia induction are excluded. For (**a**) and (**c**), *n*=38; for (**b**) and (**d**), *n*=15; for (**e**), *n*=39; for (**f**), *n*=20

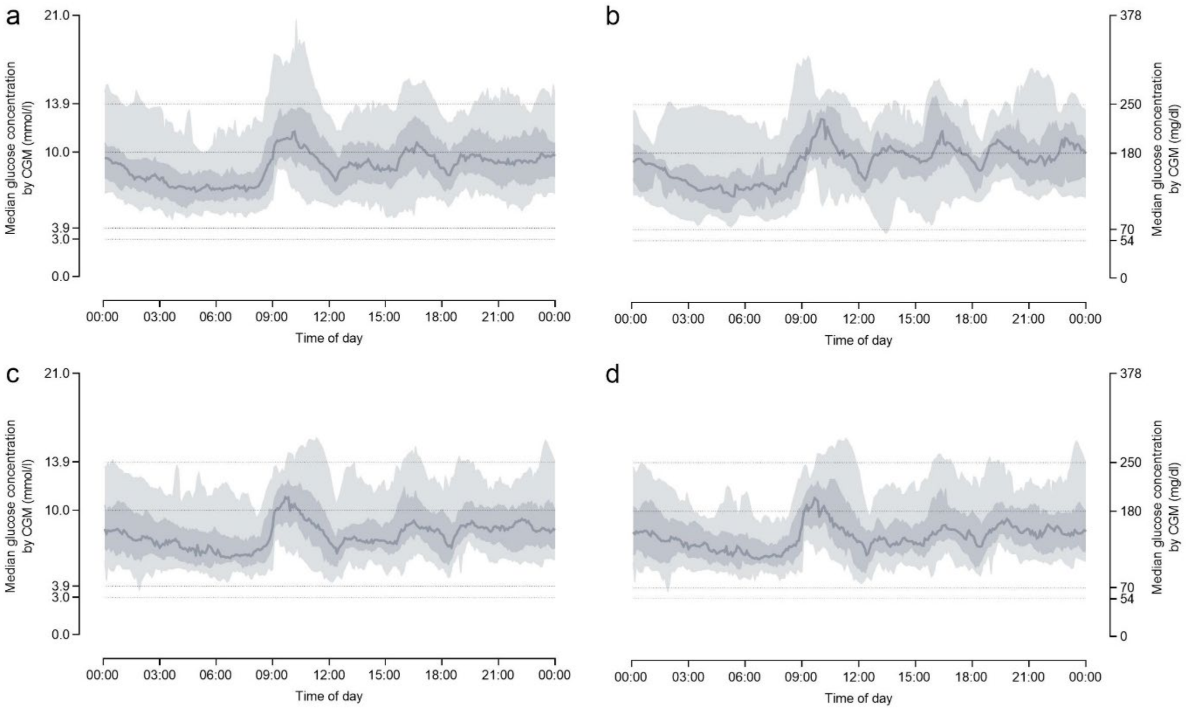

ESM Fig. 3 Median daily CGM profiles following a double or triple dose of insulin glargine U100 in all participants (**a**, **c**) and in participants with PG_{nadir} <3.0 mmol/l during hypoglycaemia induction (**b**, **d**)

(**a**, **b**) First 48 hours after double dose and (**c**, **d**) first 48 hours after triple dose are shown. Curves are median values. Dark grey bands represent the 25th to 75th percentiles and light grey bands represent the 5th to 95th percentiles. CGM data obtained during hypoglycaemia induction are excluded. For (**a**), *n*=42; for (**b**), *n*=15; for (**c**), *n*=40; for (**d**), *n*=28

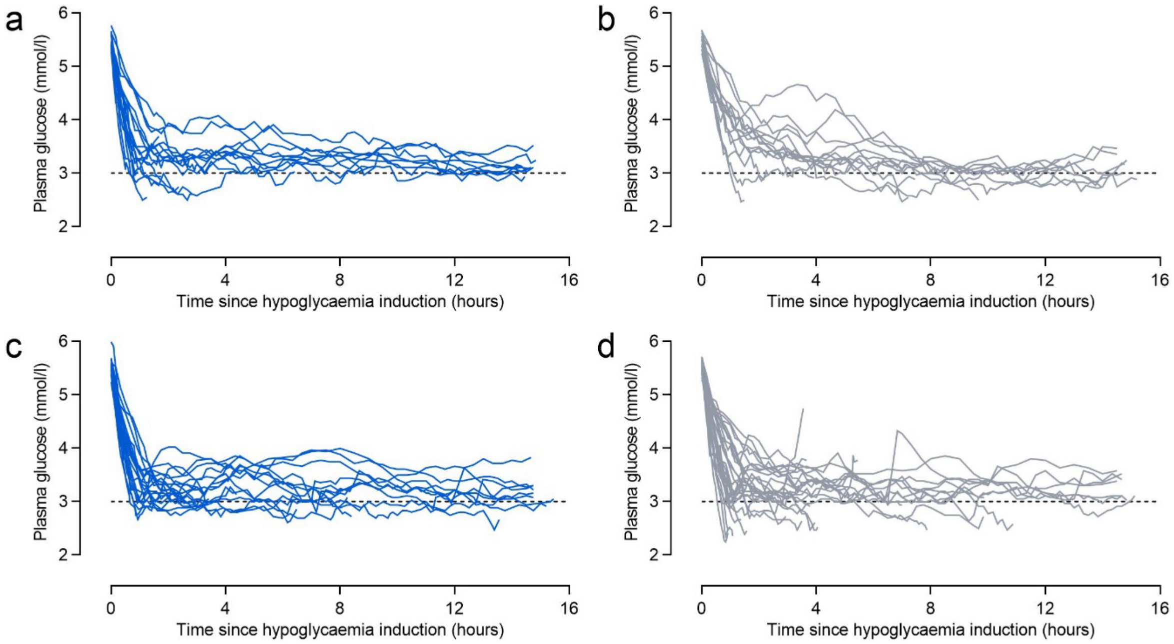

ESM Fig. 4 Individual PG profiles during development of hypoglycaemia in participants with PG_{nadir} <3.0 mmol/l

(**a**) Insulin icodec double dose, (**b**) insulin glargine U100 double dose, (**c**) insulin icodec triple dose and (**d**) insulin glargine U100 triple dose are shown. PG profiles were censored at the start of the constant glucose infusion applied to recover from hypoglycaemia. For (**a**), *n*=17; for (**b**), *n*=15; for (**c**), *n*=20; for (**d**), *n*=28

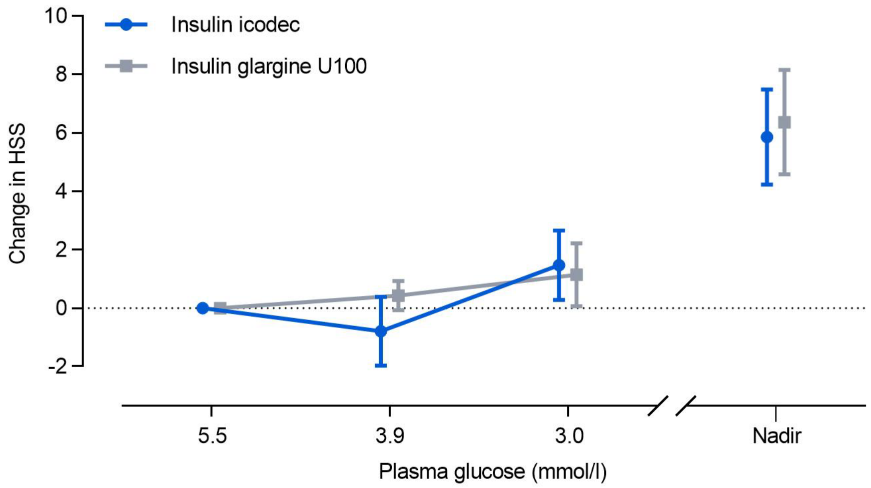

ESM Fig. 5 Change from baseline in HSS during development of hypoglycaemia following a double dose of insulin icodec or insulin glargine U100

Data are mean±SEM. n=20 for icodec except at $PG_{3.0\ mmol/l}$ (n=17), and n=19 for glargine U100 except at $PG_{3.0\ mmol/l}$ (n=15)

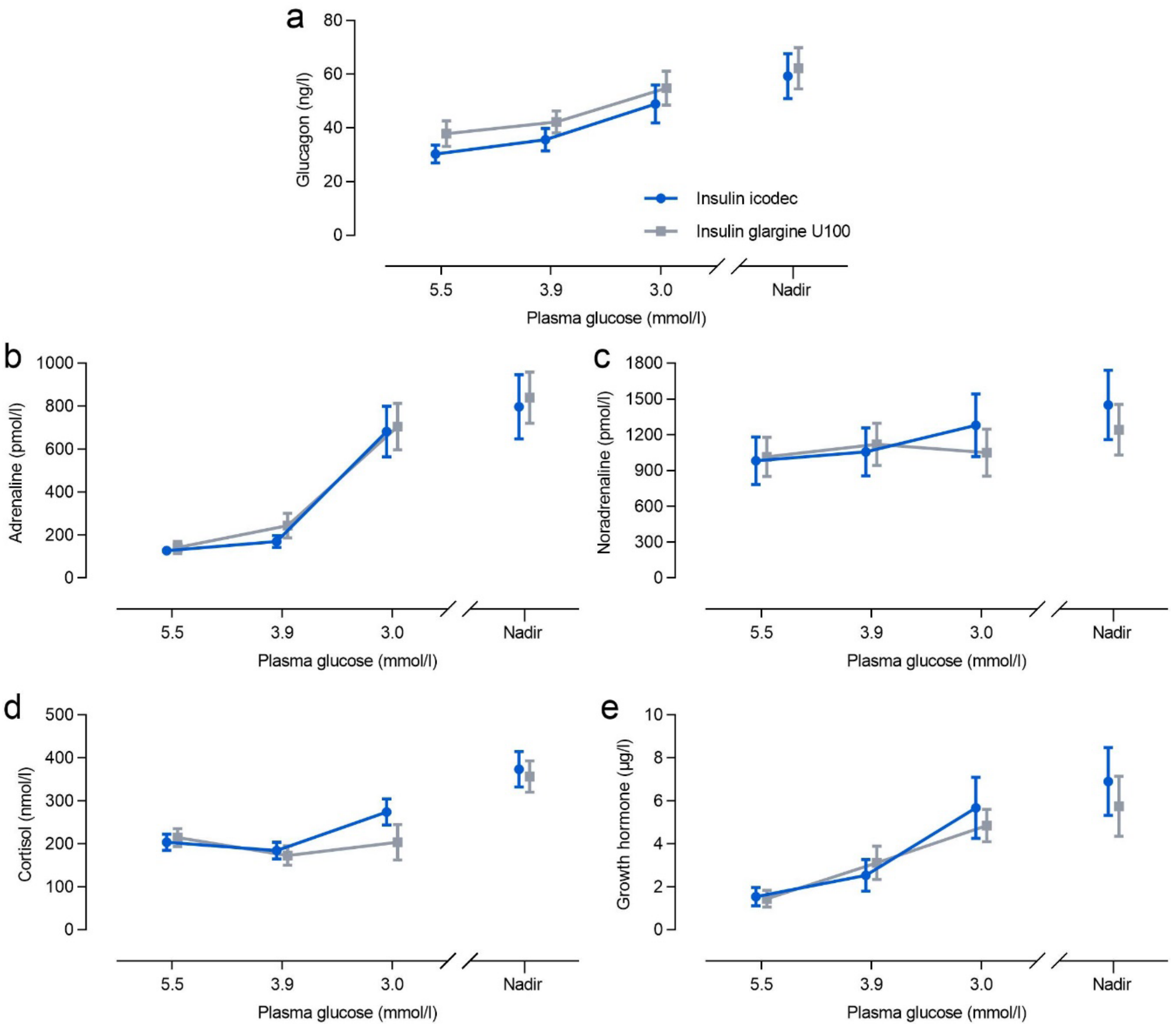

ESM Fig. 6 Counterregulatory hormone concentrations during development of hypoglycaemia following a double dose of insulin icodec or insulin glargine U100 (**a**) Glucagon, (**b**) adrenaline, (**c**) noradrenaline, (**d**) cortisol and (**e**) growth hormone levels are shown. Data are mean±SEM. For (**a**), (**d**) and (**e**), *n*=20 for icodec except at $PG_{3.0\ mmol/l}$ (*n*=17) and PG_{nadir} (*n*=19), and *n*=19 for glargine U100 except at $PG_{3.0\ mmol/l}$ (*n*=15); for (**b**) and (**c**), *n*=20 for icodec except at $PG_{3.0\ mmol/l}$ (*n*=17), and *n*=19 for glargine U100 except at $PG_{3.0\ mmol/l}$ (*n*=15)

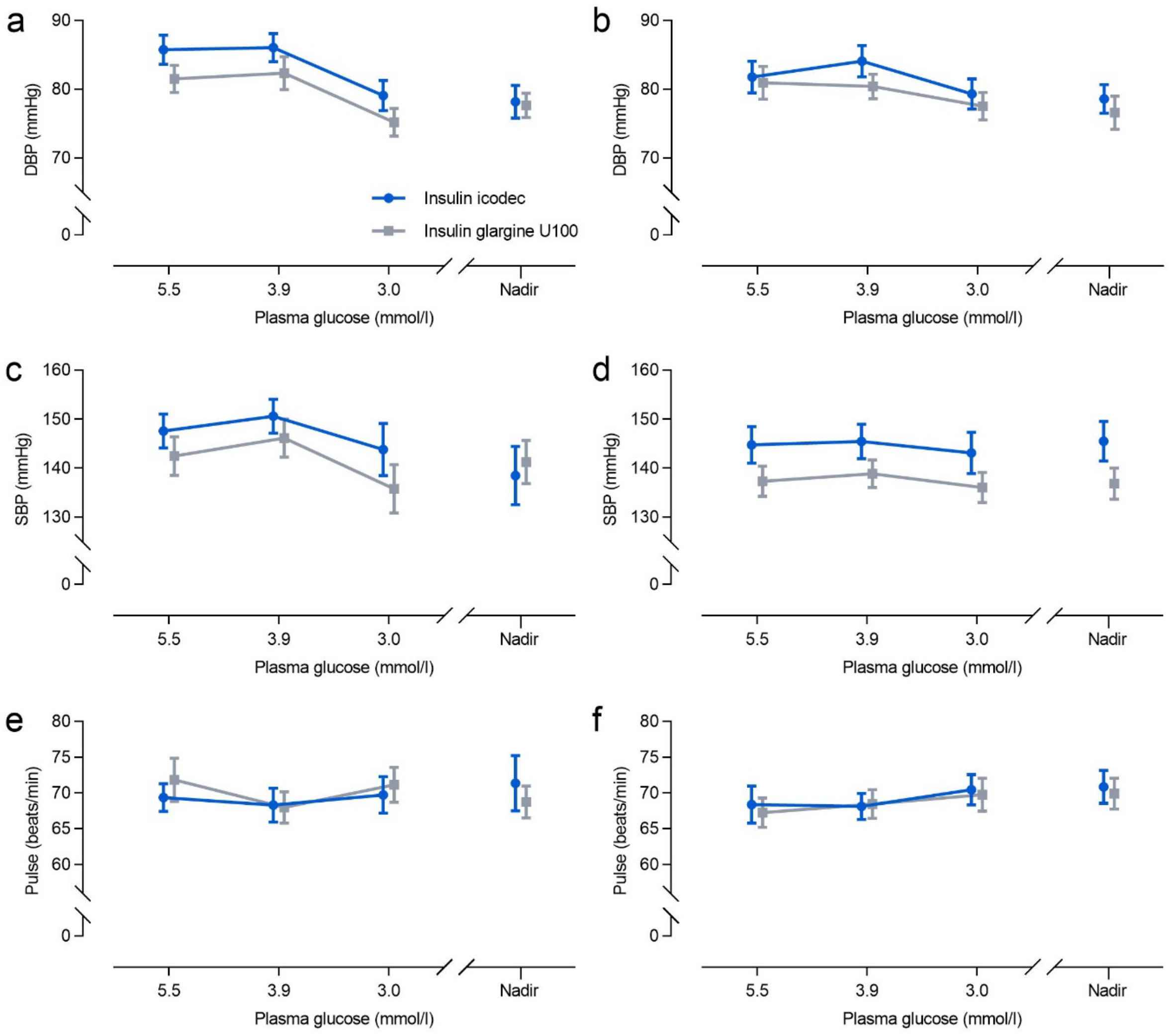

ESM Fig. 7 Vital signs during development of hypoglycaemia following a double (**a**, **c**, **e**) or triple (**b**, **d**, **f**) dose of insulin icodec or insulin glargine U100

(**a**, **b**) Diastolic blood pressure, (**c**, **d**) systolic blood pressure and (**e**, **f**) pulse are shown. Data are mean±SEM. For (**a**), (**c**) and (**e**), *n*=20 for icodec except at $PG_{3.0\ mmol/l}$ (*n*=17), and *n*=19 for glargine U100 except at $PG_{3.0\ mmol/l}$ (*n*=15); for (**b**), (**d**) and (**f**), *n*=20 for icodec, and *n*=29 for glargine U100 except at $PG_{3.0\ mmol/l}$ (*n*=28)

DBP, diastolic blood pressure; SBP, systolic blood pressure

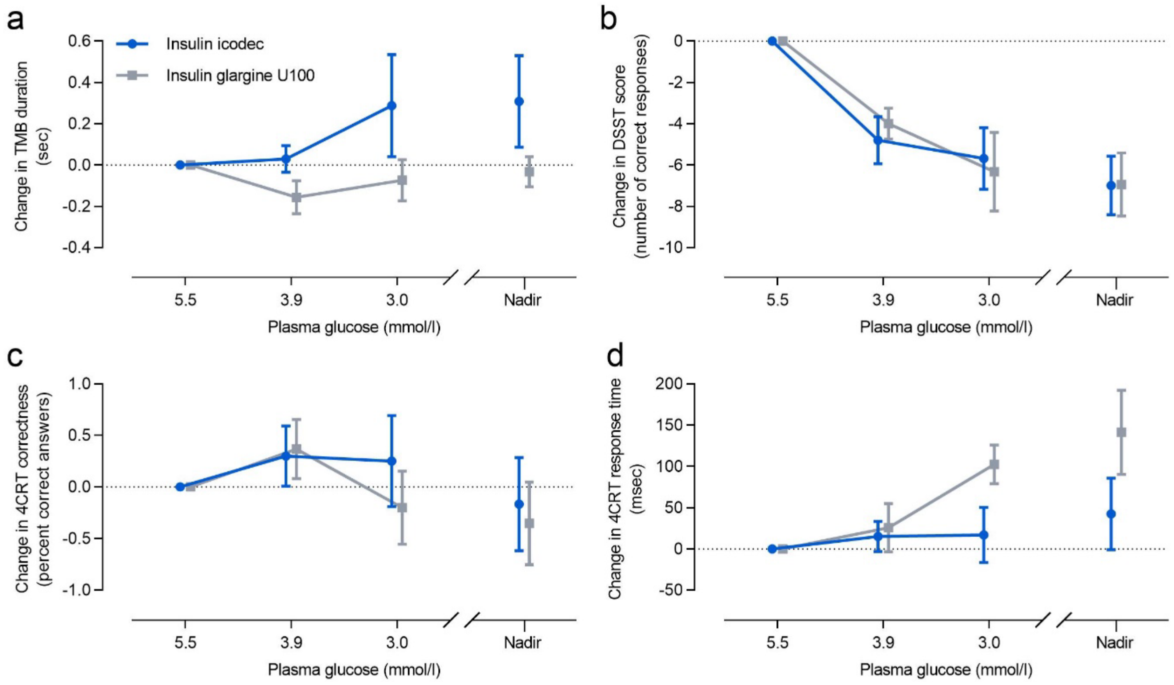

ESM Fig. 8 Change from baseline in cognitive function during development of hypoglycaemia following a double dose of insulin icodec or insulin glargine U100 Change in (**a**) TMB duration, (**b**) DSST score, (**c**) 4CRT correctness and (**d**) 4CRT response time are shown. Data are mean±SEM. For (**a**), *n*=20 at $PG_{5.5\ mmol/l}$, *n*=19 at $PG_{3.9\ mmol/l}$, *n*=16 at $PG_{3.0\ mmol/l}$ and *n*=18 at PG_{nadir} for icodec, and *n*=19 for glargine U100 except at $PG_{3.0\ mmol/l}$ (*n*=15) and PG_{nadir} (*n*=18); for (**b**) *n*=20 for icodec except at $PG_{3.0\ mmol/l}$ (*n*=16) and PG_{nadir} (*n*=18), and *n*=19 for glargine U100 except at $PG_{3.0\ mmol/l}$ (*n*=15) and PG_{nadir} (*n*=18); for (**c**) and (**d**), *n*=20 for icodec except at $PG_{3.0\ mmol/l}$ (*n*=16) and PG_{nadir} (*n*=18), and *n*=19 for glargine U100 except at $PG_{3.0\ mmol/l}$ (*n*=15) and PG_{nadir} (*n*=17)

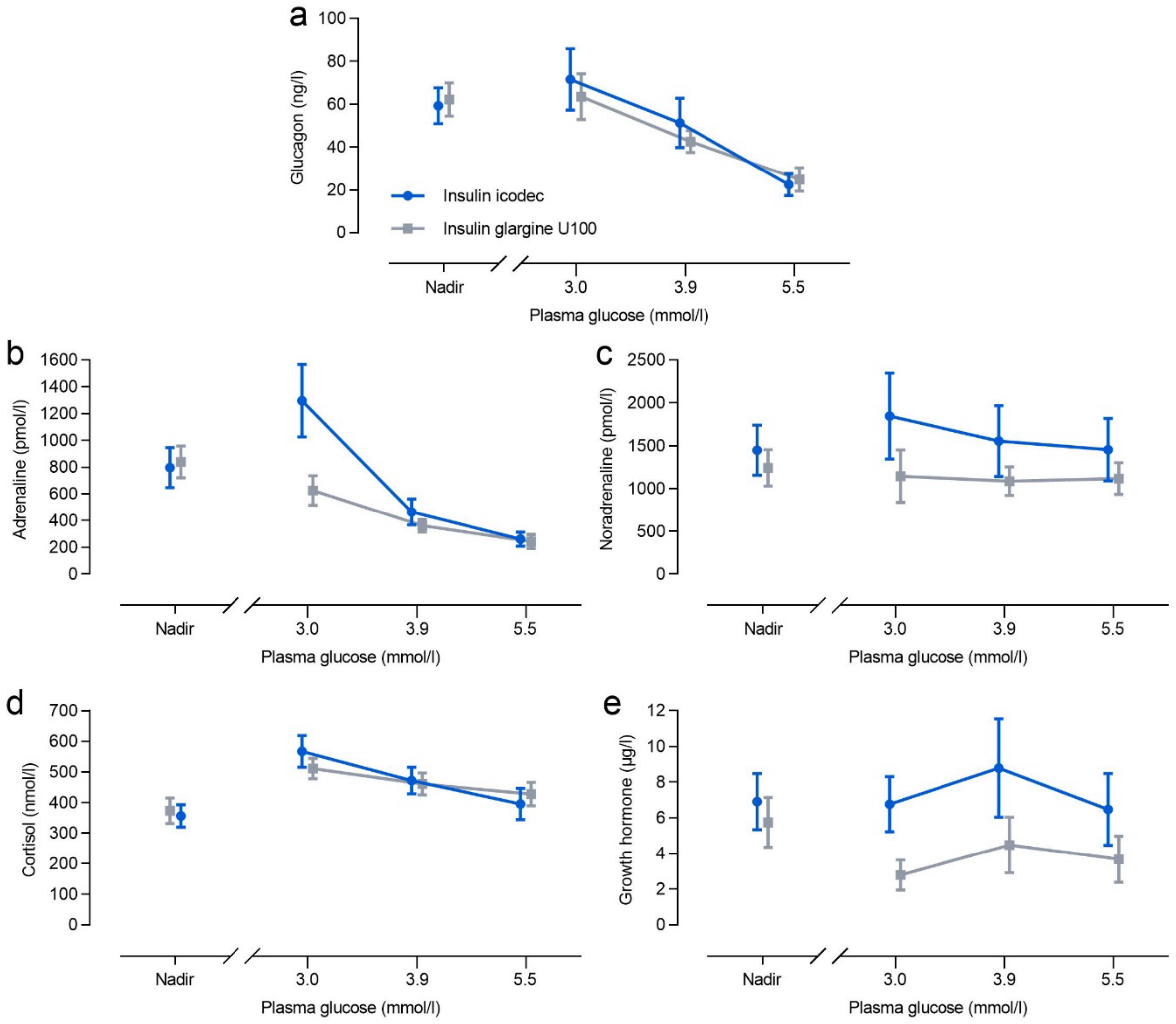

ESM Fig. 9 Counterregulatory hormone concentrations during recovery from hypoglycaemia following a double dose of insulin icodec or insulin glargine U100 (**a**) Glucagon, (**b**) adrenaline, (**c**) noradrenaline, (**d**) cortisol and (**e**) growth hormone levels are shown. Data are mean±SEM. For (**a**), (**d**) and (**e**), *n*=19 at PG_{nadir}, *n*=7 at $PG_{3.0\ mmol/l}$, *n*=11 at $PG_{3.9\ mmol/l}$ and *n*=12 at $PG_{5.5\ mmol/l}$ for icodec, and *n*=19 at PG_{nadir}, *n*=9 at $PG_{3.0\ mmol/l}$, *n*=15 at $PG_{3.9\ mmol/l}$ and *n*=16 at $PG_{5.5\ mmol/l}$ for glargine U100; for (**b**) and (**c**), *n*=20 at PG_{nadir}, *n*=7 at $PG_{3.0\ mmol/l}$, *n*=11 at $PG_{3.9\ mmol/l}$ and *n*=12 at $PG_{5.5\ mmol/l}$ for icodec, and *n*=19 at PG_{nadir}, *n*=9 at $PG_{3.0\ mmol/l}$, *n*=15 at $PG_{3.9\ mmol/l}$ and *n*=16 at $PG_{5.5\ mmol/l}$ for glargine U100

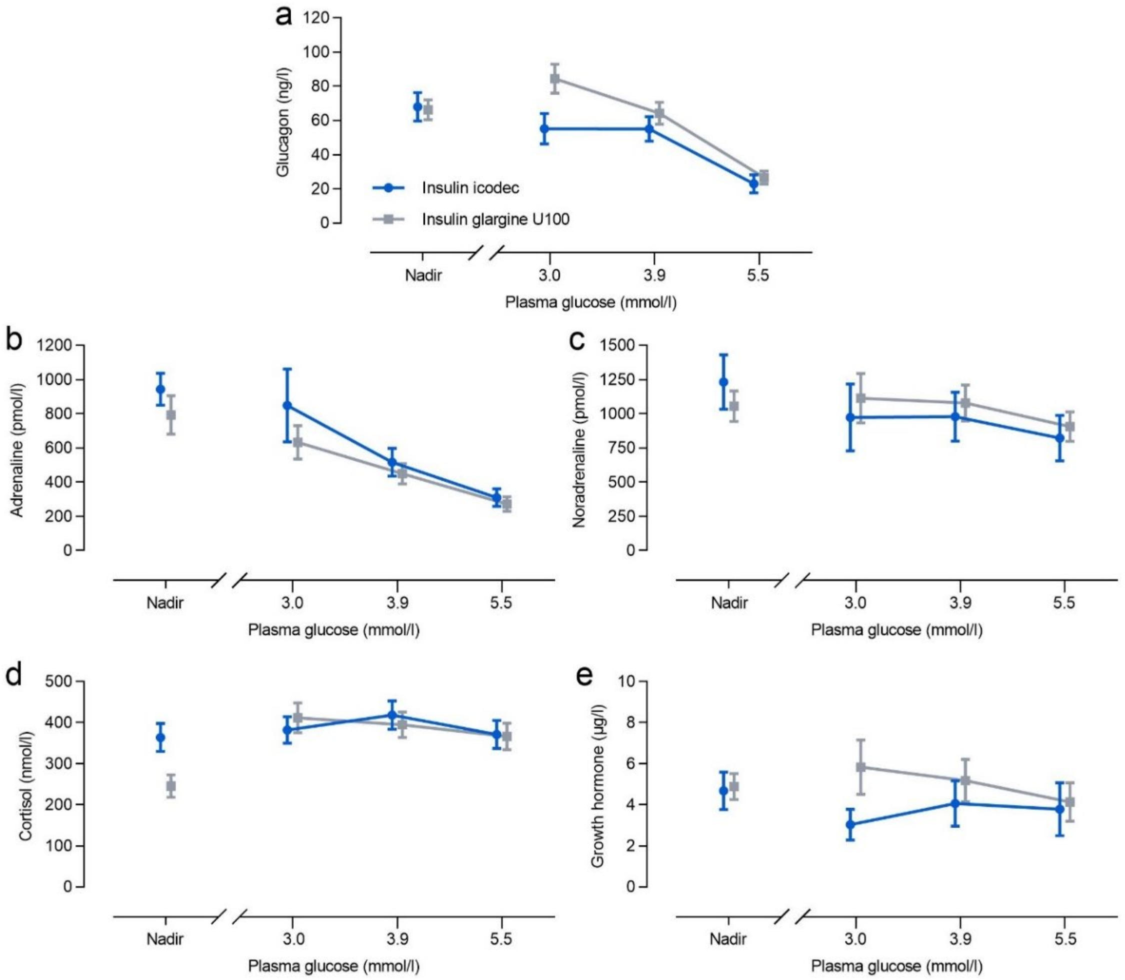

ESM Fig. 10 Counterregulatory hormone concentrations during recovery from hypoglycaemia following a triple dose of insulin icodec or insulin glargine U100

(**a**) Glucagon, (**b**) adrenaline, (**c**) noradrenaline, (**d**) cortisol and (**e**) growth hormone levels are shown. Data are mean±SEM. For (**a**) and (**b**), *n*=20 at PG_{nadir}, *n*=6 at $PG_{3.0\ mmol/l}$, *n*=13 at $PG_{3.9\ mmol/l}$ and *n*=13 at $PG_{5.5\ mmol/l}$ for icodec, and *n*=29 at PG_{nadir}, *n*=14 at $PG_{3.0\ mmol/l}$, *n*=22 at $PG_{3.9\ mmol/l}$ and *n*=23 at $PG_{5.5\ mmol/l}$ for glargine U100; for (**c**), *n*=20 at PG_{nadir}, *n*=6 at $PG_{3.0\ mmol/l}$, *n*=13 at $PG_{3.9\ mmol/l}$ and *n*=12 at $PG_{5.5\ mmol/l}$ for icodec, and *n*=29 at PG_{nadir}, *n*=14 at $PG_{3.0\ mmol/l}$, *n*=22 at $PG_{3.9\ mmol/l}$ and *n*=23 at $PG_{5.5\ mmol/l}$ for glargine U100; for (**d**) and (**e**), *n*=19 at PG_{nadir}, *n*=6 at $PG_{3.0\ mmol/l}$, *n*=13 at $PG_{3.9\ mmol/l}$ and *n*=13 at $PG_{5.5\ mmol/l}$ for icodec, and *n*=29 at PG_{nadir}, *n*=14 at $PG_{3.0\ mmol/l}$, *n*=22 at $PG_{3.9\ mmol/l}$ and *n*=23 at $PG_{5.5\ mmol/l}$ for glargine U100

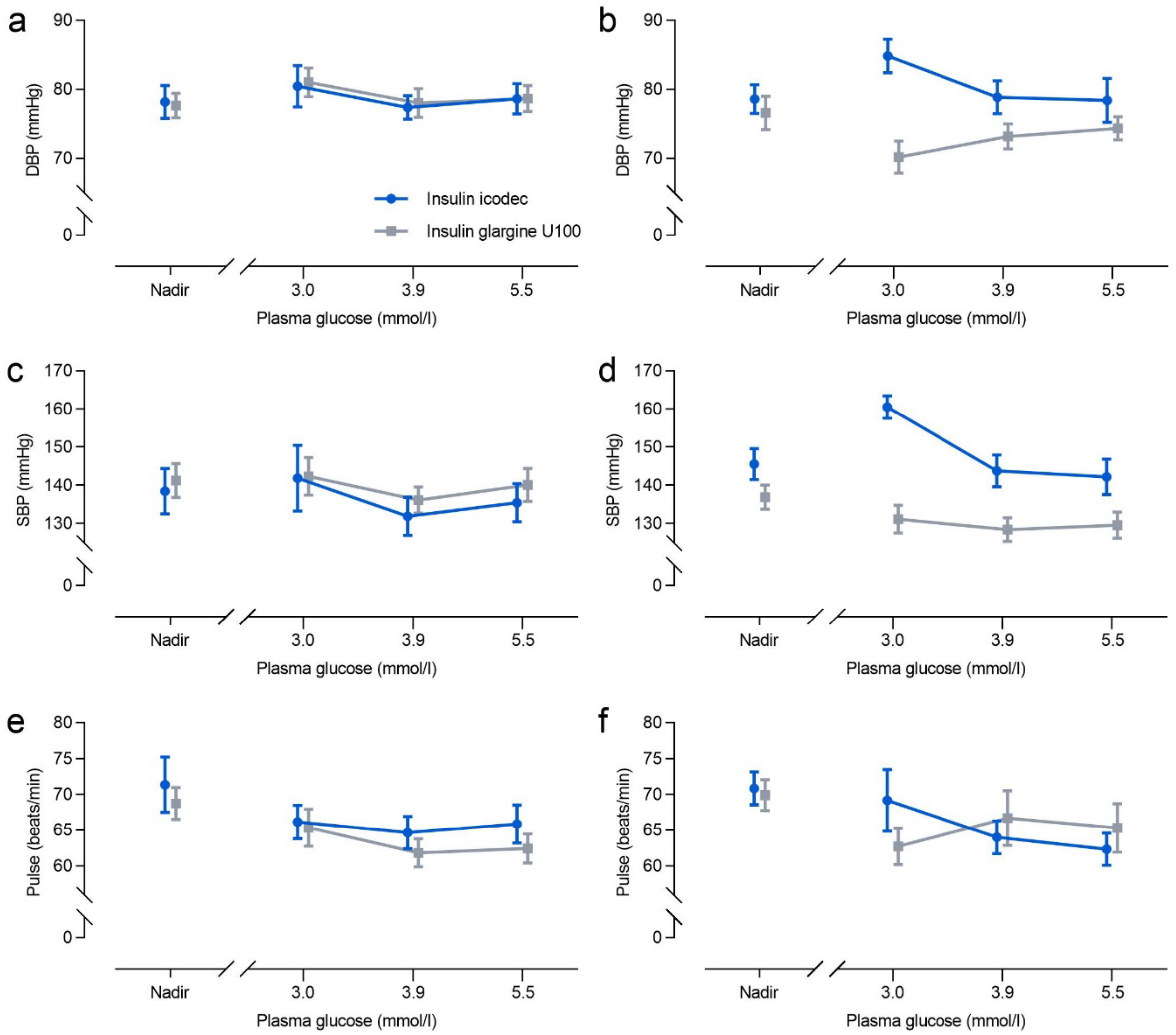

ESM Fig. 11 Vital signs during recovery from hypoglycaemia following a double (**a**, **c**, **e**) or triple (**b**, **d**, **f**) dose of insulin icodec or insulin glargine U100

(**a**, **b**) Diastolic blood pressure, (**c**, **d**) systolic blood pressure and (**e**, **f**) pulse are shown. Data are mean±SEM. For (**a**), (**c**) and (**e**), n=20 at PG_{nadir}, n=7 at $PG_{3.0\ mmol/l}$, n=12 at $PG_{3.9\ mmol/l}$ and n=13 at $PG_{5.5\ mmol/l}$ for icodec, and n=19 at PG_{nadir}, n=9 at $PG_{3.0\ mmol/l}$, n=15 at $PG_{3.9\ mmol/l}$ and n=16 at $PG_{5.5\ mmol/l}$ for glargine U100; for (**b**), (**d**) and (**f**), n=20 at PG_{nadir}, n=6 at $PG_{3.0\ mmol/l}$, n=13 at $PG_{3.9\ mmol/l}$ and n=13 at $PG_{5.5\ mmol/l}$ for icodec, and n=29 at PG_{nadir}, n=14 at $PG_{3.0\ mmol/l}$, n=22 at $PG_{3.9\ mmol/l}$ and n=23 at $PG_{5.5\ mmol/l}$ for glargine U100

DBP, diastolic blood pressure; SBP, systolic blood pressure
